# Regimes and mechanisms of transient amplification in abstract and biological neural networks

**DOI:** 10.1371/journal.pcbi.1010365

**Published:** 2022-08-15

**Authors:** Georgia Christodoulou, Tim P. Vogels, Everton J. Agnes

**Affiliations:** 1 Centre for Neural Circuits and Behaviour, University of Oxford, Oxford, United Kingdom; 2 Institute of Science and Technology Austria, Klosterneuburg, Austria; 3 Biozentrum, University of Basel, Basel, Switzerland; University of Oregon, UNITED STATES

## Abstract

Neuronal networks encode information through patterns of activity that define the networks’ function. The neurons’ activity relies on specific connectivity structures, yet the link between structure and function is not fully understood. Here, we tackle this structure-function problem with a new conceptual approach. Instead of manipulating the connectivity directly, we focus on upper triangular matrices, which represent the network dynamics in a given orthonormal basis obtained by the Schur decomposition. This abstraction allows us to independently manipulate the eigenspectrum and feedforward structures of a connectivity matrix. Using this method, we describe a diverse repertoire of non-normal transient amplification, and to complement the analysis of the dynamical regimes, we quantify the geometry of output trajectories through the effective rank of both the eigenvector and the dynamics matrices. Counter-intuitively, we find that shrinking the eigenspectrum’s imaginary distribution leads to highly amplifying regimes in linear and long-lasting dynamics in nonlinear networks. We also find a trade-off between amplification and dimensionality of neuronal dynamics, i.e., trajectories in neuronal state-space. Networks that can amplify a large number of orthogonal initial conditions produce neuronal trajectories that lie in the same subspace of the neuronal state-space. Finally, we examine networks of excitatory and inhibitory neurons. We find that the strength of global inhibition is directly linked with the amplitude of amplification, such that weakening inhibitory weights also decreases amplification, and that the eigenspectrum’s imaginary distribution grows with an increase in the ratio between excitatory-to-inhibitory and excitatory-to-excitatory connectivity strengths. Consequently, the strength of global inhibition reveals itself as a strong signature for amplification and a potential control mechanism to switch dynamical regimes. Our results shed a light on how biological networks, i.e., networks constrained by Dale’s law, may be optimised for specific dynamical regimes.

## Author summary

The architecture of neuronal networks lies at the heart of its dynamic behaviour, or in other words, the function of the system. However, the relationship between changes in the architecture and their effect on the dynamics, a structure-function problem, is still poorly understood. Here, we approach this problem by studying a rotated connectivity matrix that is easier to manipulate and interpret. We focus our analysis on a dynamical regime that arises from the biological property that neurons are usually not connected symmetrically, which may result in a non-normal connectivity matrix. Our techniques unveil distinct expressions of the dynamical regime of non-normal amplification. Moreover, we devise a way to analyse the geometry of the dynamics: we assign a single number to a network that quantifies how dissimilar its repertoire of behaviours can be. Finally, using our approach, we can close the loop back to the original neuronal architecture and find that biologically plausible networks use the strength of inhibition and excitatory-to-inhibitory connectivity strength to navigate the different dynamical regimes of non-normal amplification.

## Introduction

Recurrent network models are known to produce different types of dynamics, ranging from regular to irregular, and from transient to persistent activity [1–6]. Moulding network dynamics to resemble experimental observations usually involves changes in the network architecture, i.e., the existence of synapses and their efficacies [7–9]. With this approach, the eigenspectrum and the non-normality of the connectivity matrix are indirectly affected, and the relationship between changes in those qualities of the weight matrix and the network dynamics remain unclear. This is challenging because both the eigenspectrum and non-normality carry important information about the dynamics. The eigenspectrum, i.e., the distribution of eigenvalues in the complex plane, carries information about the stability of the network (e.g., asymptotic behaviour) [6, 8, 10], and timescale of the dynamics [4, 11]. However, the eigenspectrum alone is not sufficient to describe the transient dynamical behaviour of a network [10, 12]. The transient dynamics, and more specifically, the phenomenon of transient amplification, depends on the alignment between the eigenvectors of the connectivity matrix [10]. Importantly, the more aligned the eigenvectors are, the more *non-normal* a matrix is. The non-normality of a matrix can be assessed through the Schur decomposition, an orthogonal similarity transformation that results in an upper triangular matrix on which the eigenvalues appear along the diagonal. The Schur decomposition of a matrix **W** can be formally written as **W** = **U**(**Λ** + **T**)**U**^†^, where **U** is an unitary matrix (its columns are the orthogonal Schur modes), **Λ** is a diagonal matrix containing the eigenvalues of **W** (complex eigenvalues are represented as 2-by-2 block diagonals with the real and imaginary parts), and **T** is a strictly upper triangular matrix. The strictly upper triangular part contains information related to the interactions between the corresponding Schur modes [12]—it’s useful to note that a normal matrix has this strictly upper triangular part equal to zero because all eigenvectors are orthogonal. As such, the strength (measured by the norm [13]) of the strictly triangular part of the Schur decomposition plays an important role for the dynamics.

**Fig 1:**
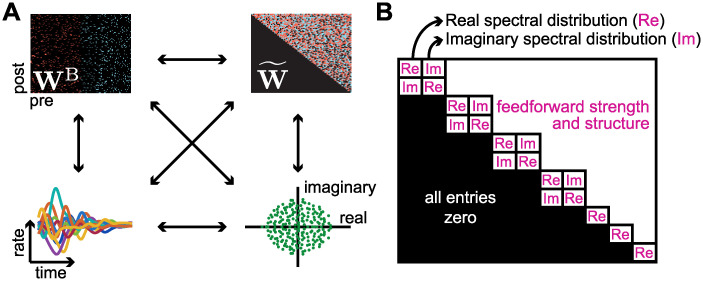
Elements explored in the manuscript. **A**, Schematic of the elements explored in this manuscript. Top left and clockwise: The connectivity matrix **W**^B^; its corresponding Schur upper triangular decomposition $\tilde{\text{W}}$; the eigenspectrum; and the induced dynamics. In **W**^B^ and $\tilde{\text{W}}$, red dots indicate positive (excitatory) connections while blue dots indicate negative (inhibitory) connections. **B**, The upper triangular matrix $\tilde{\text{W}}$ with the quantities that we alter in this manuscript in pink.

Therefore here, we consider upper triangular matrices and manipulate their spectrum and non-normality, such that their characteristics can be directly translated into dynamical properties (Fig 1A). These matrices no longer represent the neuronal connectivity, but modes of activation that are arranged in a feedforward manner [10, 12, 14] (Fig 1B). We are particularly interested in the different forms of transient amplification, a phenomenon that can resemble motor cortex activity during reaching [15–17] and also emulate long-lasting working memory dynamics [18–20]. For a network to be able to transiently amplify an input signal (i.e., initial condition), not only the eigenvectors need not be orthogonal (allowing for non-normal amplification), but also eigenvalues cannot have any real part larger than unity (allowing for the transient amplification to be followed by a decay back to baseline) [6, 10, 12]. This constrains the possible structure of the eigenspectrum. For example, when the connectivity matrix is random, the bulk of the eigenvalues is uniformly distributed on a disc centred at zero, which together with the stability constraint imposes that the radius can’t be larger than 1 [2, 6, 12]. Taking into account this effect, random weight matrices are not particularly flexible for generating distinct eigenspectrum distributions, not allowing for a rich plethora of network dynamics. Specific eigenspectrum distributions (given that the matrix is non-normal), can indeed elicit richer dynamics through either optimisation of weights taking into consideration their effect on the eigenspectrum [8] or the combination of matrices with different statistics [6, 21, 22]. However, it is still unclear how modifications in the distribution of only real or imaginary parts of the eigenspectrum change the dynamical regimes of a non-normal network which is able to transiently amplify inputs. Moreover, it’s not entirely known how these changes can be imposed to biologically realistic networks in which neurons are either excitatory or inhibitory, and neurons might not connect to themselves, i.e., without self connections.

To better understand how the manipulations in eigenspectrum and non-normality of a connectivity matrix are translated to biologically plausible networks, in this work we manipulate them directly in upper triangular matrices and then translate our main findings to biologically plausible networks. We start by defining the relationship between the network’s activity and the eigenmodes given by the Schur decomposition, showing how simple manipulations of the eigenspectrum’s imaginary diameter and real distribution can drastically affect the neuronal dynamics. We then systematically explore how the eigenspectrum’s imaginary diameter and the feedforward norm control the different regimes of transient amplification, showing that either shrinking the eigenspectrum’s imaginary diameter or increasing the feedforward norm increase amplification levels. Analysing the neuronal dynamics of these networks via the effective rank of the eigenvectors of the connectivity matrix, we find a trade-off between amplification and the underlying dimensionality of the possible dynamics of the network: networks with high (respectively low) levels of amplification produce dynamics in low (respectively high) dimensional subspaces. After a dissection of the underlying mechanisms of transient amplification using general upper triangular matrices, we consider biological constraints on the spectral distributions, and consequently, on the dynamics. Finally, we show how we can implement our findings in a biological plausible connectivity matrix with excitatory and inhibitory neurons, i.e., a matrix satisfying Dale’s law. We explore three different manipulations: lack of autapses, global inhibition, and ratio between excitatory-to-inhibitory and excitatory-to-excitatory connections. We show that increased global inhibition can lead to more amplifying dynamics due to its connections with the eigenspectrum, and that changing excitatory-to-inhibitory connections affect the eigenspectrum’s imaginary diameter.

## Results

Throughout the paper we use the following notation for the connectivity matrix: **W** for a generic connectivity matrix, $\tilde{\text{W}}$ for a matrix given in upper triangular form, and **W**^B^ for a matrix following biological constraints (Fig 1A). The dynamics of the recurrent network are defined by

$$\begin{array}{c} \tau \frac{\text{d}\text{x}(t)}{\text{d}t}=-\text{x}\left(t\right)+\text{W}f(\text{x}(t)), \end{array}$$ (1)

where **x**(*t*) is the internal state of the network at time *t*, and *x*_*i*_(*t*) can be understood as the membrane potential of the *i^th^* neuron of the network (with *i* = 1, …, *N*; *N* is the number of neurons in the network). This internal state of the neurons evolves with a characteristic time constant *τ* (fixed at *τ* = 200 ms throughout the paper) and is affected by the activity of other neurons of the network through the recurrent connections determined by **W**. Finally, the activation function, *f*(**x**(*t*)) = **r**(*t*), represents the input-output relation between the internal state, **x**(*t*), and the firing rate deviation, **r**(*t*), from the baseline activity, **r**_0_. We assume linear dynamics, **r** = *f*(**x**) = **x**, for the mathematical analysis and compare to networks with richer dynamics using a known non-linear function given by

$$\begin{array}{c} f\left(x\right)=\{\begin{array}{cc} r_{\text{min}}\text{tanh}(\frac{x}{r_{\text{min}}}) & \;\text{for}\;\;x<0\\ r_{\text{max}}\text{tanh}(\frac{x}{r_{\text{max}}}) & \;\text{otherwise,} \end{array} \end{array}$$ (2)

where *r*_min_ = 1 Hz and *r*_max_ = 4 Hz are the bounds of the sigmoid function *f*(*x*). Note that in this case, and generally used in previous works [4, 8], negative values of *r*_*i*_(*t*) means less than baseline activity (see S1 Text for a non-negative version). In the linear case, the network dynamics can be described using the eigenvalues, λ_*i*_, and eigenvectors, **v**_*i*_, of the weight matrix **W**. To quantify whether and by how much the network can amplify specific inputs, we calculate the norm of the rate vector, ‖**r**(*t*)‖, by decomposing it along the directions of the eigenvectors of **W**,

$$\begin{array}{c} \|r(t)\|=\sqrt{\sum_{k}{|r_{k}\left(t\right)|}^{2}+\sum_{k\neq j}\bar{r_{k}}\left(t\right)r_{j}\left(t\right)\left\langle v_{k},v_{j}\right\rangle }, \end{array}$$ (3)

where $\bar{r_{k}}\left(t\right)$ is the complex conjugate of *r*_*k*_(*t*) and 〈**v**_*k*_, **v**_*j*_〉 is the inner product of the complex vectors **v**_*k*_ and **v**_*j*_ (see Methods). Here, $r_{k}\left(t\right)={\hat{r}}_{k}\;\text{exp}\;(\frac{t}{\tau }\left(\lambda_{k}-1\right))$ is the solution of the system along the direction of the eigenvector **v**_*k*_, which is associated with the eigenvalue λ_*k*_ (${\hat{r}}_{k}$ is a constant, uniquely determined by the initial condition). In a stable regime, Re(λ_*k*_) < 1, ∀*k*, the system exhibits a single fixed point that represents the baseline activity. An increase of the response norm, ‖**r**(*t*)‖, with respect to the norm of the initial condition, ‖**r**(*t*_0_)‖ (here always normalised to 1), defines the phenomenon of transient amplification. A necessary condition for this to happen is the non-normality of **W**, i.e., the eigenvectors do not form an orthogonal basis [10]: 〈**v**_*k*_, **v**_*j*_〉 ≠ 0, for some *j*, *k*.

To explore regimes of transient amplification, we thus focus on matrices of the form $\tilde{\text{W}}=\Lambda +T$ (Fig 1B), with the diagonal, **Λ**, containing the eigenvalues [10, 12, 14], and the strictly upper triangular part, **T**, representing the feedforward structure between patterns of activation (see S1 Text). Note that **Λ** contains 2 × 2-blocks around the diagonal to accommodate for complex eigenvalues in real-valued matrices. The real parts of the eigenvalues are on the diagonal and the imaginary parts lie on the off-diagonal entries of the 2 × 2 blocks (Fig 1B; see Methods). We create **Λ** by sampling the real and imaginary parts of the eigenvalues from different distributions, but keeping the number of complex versus real eigenvalues constant (see Methods). The imaginary distribution needs to be symmetric with respect to zero (a condition imposed by the conjugacy of the complex eigenvalues), while the real distribution must be below 1 (and is here always set to have 0.5 as a supremum: max_λ_Re(λ) < 0.5) to ensure stability. For our analysis, we define the *spectrum norm* as the Frobenius norm of the matrix **Λ**, $\left||\Lambda |\right|=\sqrt{\sum_{k}{\left|\lambda_{k}\right|}^{2}}$ (note that the spectrum norm defined here is different than the commonly used *spectral norm* [23]). We create **T** in two different ways: from the Schur decomposition of a Stability-Optimised Circuit (SOC) [8] or sampled from a uniform distribution. A SOC is created from an initially unstable network (i.e., the initial weight matrix has some of the eigenvalues with real part greater than unity, Re(λ_*k*_) > 1 for some *k*), whose inhibitory connections are modified (optimised) so that no eigenvalue has real part greater than unity (see Methods). After optimisation, a SOC produces strong non-normal transient amplification [8]. We thus use the SOC’s corresponding feedforward structure because it is tuned to create transient amplification. We linearly scale all the elements of **T** after its structure has been fixed to vary its norm.

The subsequent sections are organised as follows. First, we explore the effects of changing the distribution of the eigenspectrum’s imaginary and real parts. We then examine the influence of the spectrum and feedforward norms, ||**Λ**|| and ||**T**||, respectively, for different regimes of transient amplification, analysing the dimensionality of the dynamics in such regimes. Finally, we link some of the findings from abstract to biological networks through manipulations in a biologically realistic weight matrix that satisfies Dale’s law.

### Shrinking the imaginary distribution increases amplification

**Fig 2:**
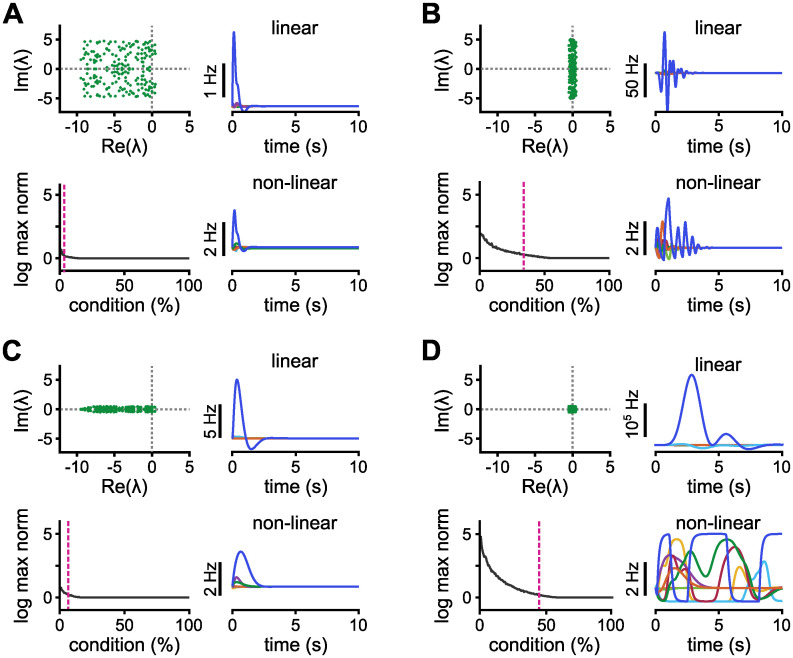
Eigenspectra and dynamics of the corresponding networks. **A–D**, Four cases of eigenspectra and dynamics of the corresponding network of size *N* = 200. In each panel, clockwise: The spectrum; linear dynamics; non-linear dynamics; the logarithm of the maximum norm of the firing rate per initial condition. The same initial condition that elicits the maximum norm is used for both linear and non-linear dynamics. Pink dotted line indicates the percentage of conditions whose norm is amplified by at least 50%. The feedforward structure is taken from a stability-optimised circuit [8] and its Frobenius norm is fixed to 75. Real and imaginary parts follow an uniform distribution with diameters *d*_im_ and *d*_re_, respectively. **A**, When *d*_im_ = *d*_re_ = 10, only 1% (2 out of 200) of the conditions are slightly amplified. **B**, When *d*_im_ = 10 and *d*_re_ = 1, the system is capable of more amplification. **C**, Here, *d*_im_ = 1 and *d*_re_ = 10, surprisingly creating more amplification compared to the case shown in panel A. **D**, When *d*_im_ = *d*_re_ = 1, the system amplifies almost half of the initial conditions. The dynamics, given an initial condition of norm 1, reach the value of ∼ 10^5^ Hz in the linear case and consequently long-lasting dynamics in the non-linear case.

We start our investigation of how the eigenspectrum affects the dynamics by drawing both real and imaginary parts from uniform distributions with diameters *d*_im_ and *d*_re_, respectively (Fig 2A–2D, top left). To quantify the dynamical response of the network, we find an orthogonal basis of initial conditions of the linear network that elicit maximum amplification, $I_{B}=\left\{a_{1},\ldots ,a_{N}\right\}$, ordered according to their *evoked energy* [8], E(a): if **x**(*t* = 0) = **a**, ||**a**|| = 1, then $E\left(a\right)=\frac{2}{\tau }\int_{0}^{\infty }{\left||\text{x}(t)|\right|}^{2}\text{d}t$. We first calculate the initial condition **a**_**1**_ that maximises E(a), following an iterative calculation of the subsequent orthogonal initial condition, **a**_**i**_, that maximise E(a) in the subspace orthogonal to the previously calculated initial conditions, {**a**_**1**_, …, **a**_**i**−**1**_}. To make sure that the evoked energy is due to an amplified response rather than merely a slower exponential decay, we compute the maximum value of the norm of the firing rate vector, for all vectors in $I_{B}$ (Fig 2A–2D, bottom left).

With broad distributions, the system can slightly amplify a few conditions (Fig 2A). When the range of the real-part distribution is decreased and the real parts are pushed towards 0.5, the resulting network produces stronger amplification (Fig 2B). This can mainly be attributed to the fact that the eigenvalues have now larger real parts and hence longer decay envelopes. A longer decay envelope allows for the network dynamics to undergo a larger proportion of a full period of the underlying oscillation without damping of the maximum amplitude, revealing the hidden feedforward structure, and thus increasing the maximum norm. Indeed, clustering away from 0.5 leads to less amplification (explored in the next section).

More surprisingly, shrinking, instead, the imaginary distribution also leads to more amplification (Fig 2C), and shrinking both distributions produces very large amplification that in the non-linear case lasts for a long time (longer than 10 seconds), approximating timescales of working memory dynamics (Fig 2D), previously known to arise through spectral abscissas near the stability line. Additionally, the percentage of conditions that are amplified is considerably increased, i.e., the ability of such a network to amplify orthogonal initial conditions is enhanced. Note that splitting and clustering the (positive and negative) imaginary parts away from zero gives rise to slightly different amplification regimes that also depend on the linearity of the system (S1 Fig). From these examples (Fig 2 and S1 Fig) it is clear that varying the diameter and position of the centre of the distribution of both imaginary and real parts of the eigenspectrum play distinct roles in the levels of amplification of a network undergoing transient amplification.

### Manipulating the spectrum and the feedforward norms

**Fig 3:**
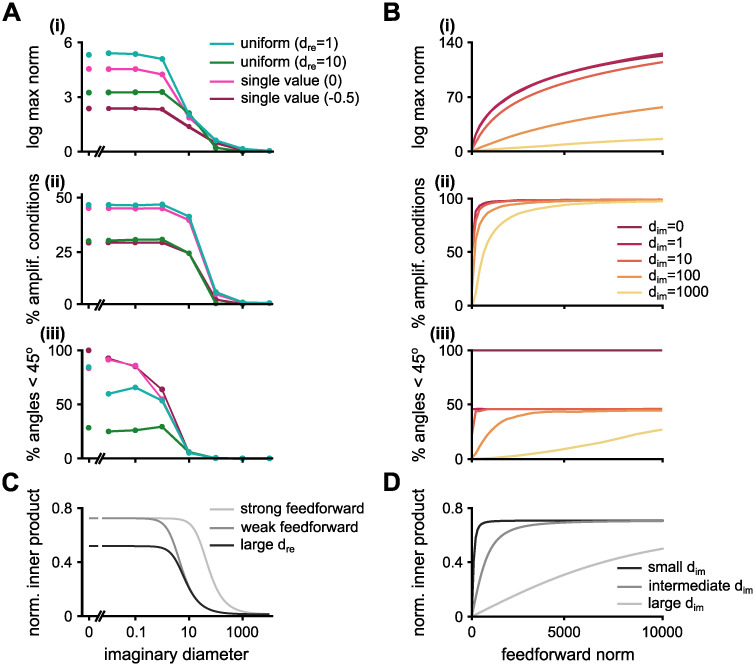
Effects of manipulating the spectrum and the feedforward norms. **A**, Maximum response norm for the preferred initial condition **(i)**, percentage of directions whose norm is amplified more than 50% **(ii)**, and the percentage of angles (between pairs of eigenvectors) that are less than 45° **(iii)**. Every line is a function of the imaginary diameter. We plot four real distributions. Light green: a uniform distribution in which all real parts are distributed uniformly in the interval (−0.5, 0.5). Dark green: a uniform distribution in which all real parts are distributed uniformly in the interval (−9.5, 0.5). Light pink: a single valued real distribution in which all real parts are equal to zero. Dark pink: a single valued real distribution in which all real parts are equal to −0.5. In all cases the network size is *N* = 200 and the feedforward Frobenius norm is fixed at 75. *d*_re_ indicates the diameter of the uniform distribution of the eigenspectrum’s real part. **B**, Same as panel A, but plotted as a function of the feedforward Frobenius norm. Different colours correspond to 5 different spectra; all spectra have fixed single-valued real distributions (equal to zero) and different imaginary diameters. *d*_im_ indicates the diameter of the uniform distribution of the eigenspectrum’s imaginary part. **C**, Normalised inner product between vectors from a simplified 3-by-3 upper triangular matrix (Eq 4) as a function of the imaginary diameter (*β* in Eq 4) for three conditions: “strong feedforward” (*ϕ*_norm_ = 30 and *α* − *γ* = −0.3); “weak feedforward” (*ϕ*_norm_ = 3 and *α* − *γ* = −0.3); and “large d_re_” (*ϕ*_norm_ = 3 and *α* − *γ* = −3). **D**, Same as panel C, but plotted as a function of the feedforward norm for three different conditions: “small d_im_” (*β* = 100 and *α* − *γ* = 0); “intermediate d_im_” (*β* = 1000 and *α* − *γ* = 0); and “large d_im_” (*β* = 10000 and *α* − *γ* = 0).

When we study the effects of the imaginary and real distributions more systematically, we find that the shape of the real distributions affects the levels of amplification, but has minimum effect on how amplification changes with the imaginary distribution (Fig 3Ai and 3Aii and S2(A) Fig). Amplification emerges from the non-normality of **W**, which can be partly quantified by the angles between the eigenvectors (Eq 3); if more pairs have overlaps, the matrix will be more non-normal. The imaginary distribution changes the geometry of the eigenvectors (Fig 3Aiii), providing a mechanism for its drastic effect on the amplification in these networks (Fig 3Ai and 3Aii). This is a surprising effect given that we do not alter the feedforward norm, ‖**T**‖, i.e., the Frobenius norm of the strictly upper triangular part of $\tilde{\text{W}}$, nor the decay envelopes at all (see S2(B) Fig and S1 Text for variations of feedforward structures).

The feedforward norm is more directly linked to the non-normality [10], and as expected, it increases both the norm of the maximum response (Fig 3Bi), and the percentage of amplified conditions (Fig 3Bii), for larger values. The percentage of eigenvector pairs with small angles also grows with increasing feedforward norm strength (Fig 3Biii). Interestingly, there is a saturating point of eigenvector pairs aligned with angles smaller than 45° that depends on the imaginary distribution. Once the number of pairs saturates, increased amplification may be associated with an increase in the matrix norm, ||**W**||, or further alignment of these eigenvector pairs (even smaller angles).

To get an intuition of the mechanisms behind the changes in amplification levels we analysed a 3-by-3 upper triangular matrix with one purely real eigenvalue, λ_1_ = *γ*, and two complex eigenvalues, $\lambda_{2}=\bar{\lambda_{3}}=\alpha -i\beta$. Two out of the three eigenvectors of this matrix are orthogonal, 〈**v**_2_, **v**_3_〉 = 0. The inner product of the non-orthogonal eigenvectors is given by (see Methods for details)

$$\begin{array}{c} |\frac{\left\langle v_{1},v_{2}\right\rangle }{\|v_{1}\left\|\right\|v_{2}\|}|=|\frac{\left\langle v_{1},v_{3}\right\rangle }{\|v_{1}\left\|\right\|v_{3}\|}|=\frac{\phi_{\text{norm}}}{\sqrt{2[\phi_{\text{norm}}^{2}+(\alpha -\gamma {)}^{2}+\beta^{2}]}}, \end{array}$$ (4)

where *ϕ*_norm_ is the feedforward norm. The result from the simplified 3-by-3 matrix follows the same trends as the simulations (Fig 3C and 3D). The overlap between the eigenvectors increases as the imaginary part of the eigenvalues, *β*, decreases—equivalent to shrinking the imaginary diameter in the large upper triangular connectivity matrix, $\tilde{\text{W}}$. The eigenspectrum’s real elements, *α* and *γ*, have a similar effect: the larger their difference the more the eigenvectors are aligned. Interestingly, when *α* = *γ*, the real distribution has no influence over the overlap between the eigenvectors, such as seen when we decrease the single real-valued distribution (S2(A) Fig, bottom)—the decrease in amplification levels is mostly due to the faster decay times when decreasing the single real-value distribution (S2(A) Fig, top). Because of the square, the larger the absolute value of *α* − *γ* (negative or positive) the less the eigenvectors are aligned, which we confirm with a large upper triangular matrix, $\tilde{\text{W}}$, varying the diameter of the eigespectrum’s real distribution while keeping its maximum value at max_λ_Re(λ) = 0.5 (S2(C) Fig). The eigenspectrum’s real distribution may affect the level of amplification in two ways: it changes the asymptotic behaviour (decay times) and the eigenvector alignment. These results give a broad intuition for the distinct contribution of the eigenspectrum’s imaginary diameter and feedforward norm, but we still do not exactly know how the dynamics of such networks evolve. Thus, we next study the relative directions of the eigenvectors in state-space.

### The geometry of output trajectories

If most eigenvectors are pointing in similar directions, the dynamics will be biased towards these directions too (Eq 3). This does not mean that **W** or the eigenvector matrix **V** are not full rank—on the contrary, they almost always are. What it means is that, in order to quantify the global eigenvector geometry, we have to use the effective rank of **V**. The effective rank of **V** measures the average number of significant dimensions in its range, and is formally defined as the exponential of the spectral entropy of its normalised singular values [24]. Specifically, if *σ*_1_, *σ*_2_, ⋯, *σ*_*N*_ are the singular values of **V**, and $p_{i}=\frac{\sigma_{i}}{{\left\|\sigma \right\|}_{1}}$, with ${\left\|\sigma \right\|}_{1}=\sum_{k=1}^{N}\left|\sigma_{k}\right|$, then

$$\begin{array}{c} \text{erank}\left(V\right)=\text{exp}[H(p_{1},\cdots ,p_{N})], \end{array}$$ (5)

where *H*(*p*_1_, ⋯, *p*_*N*_) is the Shannon entropy, i.e., $H\left(p_{1},\cdots ,p_{N}\right)=-\sum_{k=1}^{N}p_{k}\text{log}p_{k}$.

**Fig 4:**
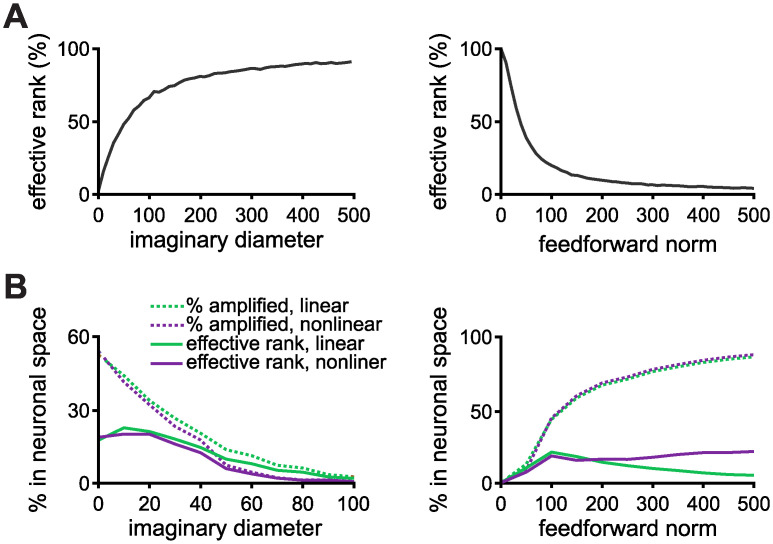
Geometry of output trajectories. **A**, The effective rank of the eigenvector matrix **V** of $\tilde{\text{W}}$ as a function of the imaginary diameter (left) and the feedforward norm (right). **B**, Amplified directions and effective rank of the matrix P (see text) in the linear and nonlinear cases as a function of the imaginary diameter (left) and the feedforward norm (right). The feedforward structure is random from a uniform distribution, and the real distribution is uniform on (−0.5, 0.5). In all cases the network size is *N* = 200. The feedforward Frobenius norm is fixed at 75 for the plots with varying imaginary diameter. The imaginary diameter is fixed at 20 for the plots with varying feedforward norm.

The effective rank of **V** is indeed small in the highly amplifying regimes (Fig 4A), revealing an underlying duality between amplification and output dimensionality. The consequence for the dynamics is that, even though the system may amplify many initial conditions, they nevertheless evolve in the same low dimensional subspace [22]. To identify the dimensionality of this subspace we compute the effective rank of the matrix P which is constructed as follows: the *j^th^* column of P is the first principal vector of the dynamics, given the *j^th^* amplified initial condition of the $I_{B}$ basis. We find that there is a discrepancy between the number of amplified directions and effective rank when the system produces large amplifications (Fig 4B; see S3 Fig for results with larger number of principal components). This suggests that the dynamical responses evoked by orthogonal initial conditions evolve in the same subspace, indicating that any added noise will be amplified in the same subspace and that different initial conditions could potentially lead to similar linear readouts (see ref. [6] for capacity estimates of transient amplifying networks). There is thus a trade-off between the number of orthogonal amplified conditions and the noise robustness of the system. The effective rank of **V** is preserved in a recurrent network that is a rotated version of an upper triangular one (S4(A) Fig) while the effective rank of the connectivity matrices differ (S4(B) Fig). Similarly, the discrepancy between the number of amplified directions and effective rank is preserved for the rotated (recurrent) weight matrix (S4(C) Fig; compare to Fig 4B), which highlights the robustness of our method to describe the geometry of the network dynamics.

**Fig 5:**
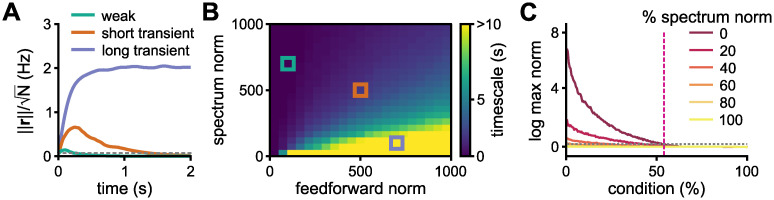
Amplification regimes. **A**, Representative examples of non-normal amplification defined by the timescale of the transient response of the nonlinear network—period, Δ*t*, for which ‖**r**(*t*)‖ ≥ 1: “weak” (Δ*t* ≤ 500 ms); “short transient” (500 < Δ*t* < 2000 ms); and “long transient” (Δ*t* ≥ 2000 ms). Grey dotted line indicates ‖**r**‖ = 1. **B**, Timescale of the response in the nonlinear network (as in panel A), parametrised by the norms of the spectrum and feedforward structure. Yellow indicates timescale longer than 10 seconds. Boxes correspond to the values used for the plots in panel A (colour coded): (feedforward norm, spectrum norm) = (100, 700), (500, 500), and (700, 100) for weak, short transient, and long transient, respectively. **C**, Maximum norm of the dynamical response per initial condition for different percentages of the norm assigned to the spectrum, ranging from a matrix whose entire norm is assigned to the spectrum (yellow; 100% case, normal matrix) to a matrix whose entire norm is assigned to the feedforward part (dark red; 0% case, nilpotent matrix). The network size is *N* = 200 in all panels. Both eigenspectrum and feedforward structures are random uniform.

To further describe the system, we use the timescale of the transient amplification, i.e., period, Δ*t*, for which ‖**r**(*t*)‖ ≥ 1 for the nonlinear network (Fig 5A and S5 Fig). This timescale varies continuously as a function of the norms of the eigenspectrum and the feedforward structure (Fig 5B and S6(A)–S6(F) Fig). Importantly, the dynamics evoked in each of these regimes—defined here by the transient amplification period—lie in different subspaces. For very short periods (e.g., Δ*t* ≤ 500 ms; Fig 5A “weak”), the eigenvectors are effectively orthogonal to each other but span the entire output space equally. For short transient periods (e.g., 500 < Δ*t* < 2000 ms; Fig 5A “short transient”), there is a good balance between amplification of orthogonal inputs and diversity in the responses. For long transient periods (e.g., Δ*t* ≥ 2000 ms; Fig 5A “long transient”), many initial conditions are amplified but the responses lie in the same low-dimensional subspace. This result is well explained by the alignment of the eigenvectors of the simplified 3-by-3 upper triangular matrix (Eq 4): larger feedforward norm or smaller eigenspectrum norm result in eigenvectors being more aligned (S6(G)–S6(I) Fig). Indeed, when we fix the norm of **W**, and distribute a—continuously decreasing—percentage of this norm on the diagonal and the rest on the feedforward structure, the network transitions from weakly to strongly amplifying (Fig 5C). Thus, it’s the relation between the diagonal (representing the spectrum) and feedforward parts of the matrix that shapes the dynamics of the network.

### Towards biologically realistic networks that satisfy Dale’s law

Up to here we explored different regimes of transient amplification in networks defined by an upper triangular connectivity matrix. This allowed us to have precise control over both eigenspectrum and feedforward structure of the connectivity matrix. However, these abstract networks do not represent biologically realistic neural networks, especially because of Dale’s law, i.e., neurons are either excitatory or inhibitory. In an upper triangular connectivity matrix, $\tilde{\text{W}}$, the feedforward structure (and consequently feedforward norm) is given by the elements of its strictly upper triangular part, while the eigenspectrum (and consequently spectrum norm) is given by the elements of its diagonal. However, both the eigenspectrum and the feedforward structures are not trivially manipulated in a biological connectivity matrix, **W**^**B**^. As a last application, we thus explore how to navigate the regimes of transient amplification in biological networks (i.e., satisfying Dale’s law) based on our results from upper triangular matrices. First, we consider the effect of the absence of self loops in the connectivity matrix. We then focus on the indirect manipulation of the spectrum norm and the distribution of eigenvalues via modifications of global inhibition and the indirect manipulation of the eigenspectrum’s imaginary diameter via modifications of the ratio between excitatory-to-inhibitory and excitatory-to-excitatory connections, respectively.

In the simulations of networks satisfying Dale’s law, half of the neurons are chosen to be excitatory (only positive output weights) and the other half to be inhibitory (only negative output weights) [2, 8, 12]. For additional biological plausibility, the connections are sparse, i.e., elements are set to zero following an uniform distribution. We thus have a set of constraints to fulfil: *(i)* stable asymptotic dynamics (lim_*t*→∞_ ‖**r**(*t*)‖ = 0, i.e., max_λ_Re(λ) < 1); *(ii)* transient amplification (‖**r**(*t*)‖ > ‖**r**(0)‖ for *t*_0_ < *t* < *t*_0_ + Δ*t*, with *t*_0_ ⩾ 0 and Δ*t* > 0); and *(iii)* neurons are excitatory or inhibitory. To do so we build biological weight matrices, **W**^**B**^, with the algorithm from Stability-Optimised Circuits (SOC) [8] (see Methods), which fulfils all constraints mentioned above. The SOC algorithm optimises inhibitory connections to ensure that no eigenvalue has real part greater than *α*_max_, *α*_max_ < 1 (max_λ_Re(λ) < *α*_max_). Moreover, it allows for additional constraints to be implemented, such as the level of global inhibition, and whether neurons have autapses, which we use to translate the results from upper triangular matrices, $\tilde{\text{W}}$, to biological matrices, **W**^**B**^.

#### The absence of neuronal self loops shrinks the real distribution

Experimental evidence supports the existence of autapses [25, 26], yet it is common for modelling work to impose no self connections [27, 28]. As a starting point, we thus analysed the consequence of the lack of self connections in networks, i.e., networks without autapses. When neurons are not structurally connected to themselves, the trace of the weight matrix of such a network is equal to zero. This unfolds as follows:

$$\begin{array}{c} \text{Tr}\left({\text{W}}^{B}\right)=\sum_{i=1}^{N}\lambda_{i}=i\sum_{i=1}^{N}\text{Im}\left(\lambda_{i}\right)+\sum_{i=1}^{N}\text{Re}\left(\lambda_{i}\right)=0. \end{array}$$ (6)

Given that $\sum_{i=1}^{N}\text{Im}\left(\lambda_{i}\right)=0$ due the conjugacy of the eigenvalues, the weight matrix **W**^**B**^ without self loops has $\sum_{i=1}^{N}\text{Re}\left(\lambda_{i}\right)=0$. This, together with the stability constraint, max_λ_Re(λ) = *α*_max_ < 1, bounds the real distribution from below and above, restricting it to a limited diameter that is less than *α*_max_*N*. The maximum range for the real distribution is bounded by −*α*_max_(*N* − 1) and *α*_max_, when all real parts but one—defined here as the outlier—are equal to *α*_max_ and the outlier is equal to −*α*_max_(*N* − 1). This observation explains why the spectrum of a stability-optimised circuit [8], which doesn’t have self loops, has an elongated shape along the imaginary axis after optimisation. Not only the positive but also the negative real parts of the eigenspectrum are pushed towards the stability line after optimisation, with the exception of the outlier, which has a large negative value due to the non-self loops constraint and inhibition dominating over excitation [8]. The zero trace condition (particularly $\sum_{i=1}^{N}\text{Re}\left(\lambda_{i}\right)=0$) is necessary but not a sufficient condition for the absence of self loops, yet it provides an intuition for its effect on networks defined by upper triangular matrix that can be translated to biological networks.

**Fig 6:**
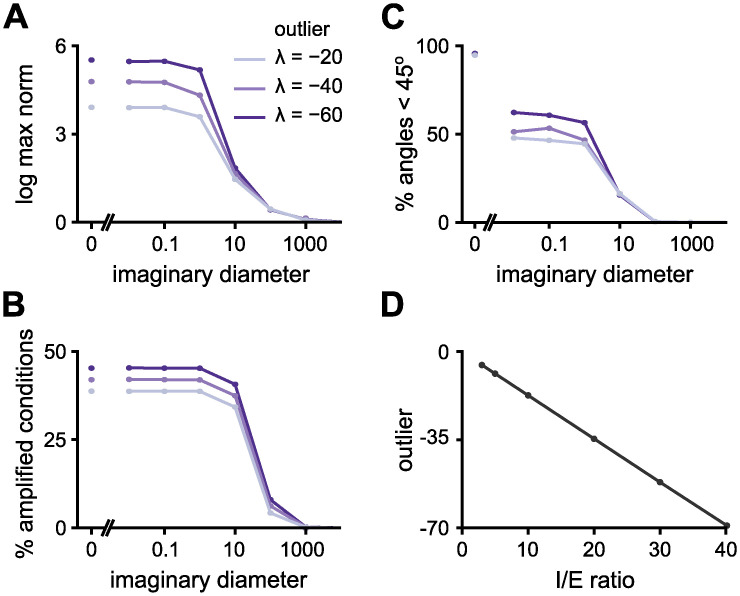
A large negative outlier increases amplification in upper triangular matrices without self loops and is proportional to *I*/*E* ratio in networks satisfying Dale’s law. **A**, Maximum response norm for the preferred initial condition as a function of the imaginary diameter using upper triangular connectivity matrices with the zero trace condition and different outliers (coloured coded). The network size is *N* = 200 and the feedforward Frobenius norm is set to 75 in all cases. **B**, Percentage of directions whose norm is amplified more than 50% as function of the imaginary diameter as in panel A. **C**, The percentage of angles, between pairs of eigenvectors, that are less than 45°, as a function of the imaginary diameter as in panel A. **D**, Position of the outlier as a function of the *I*/*E* ratio for a network with 100 excitatory and 100 inhibitory neurons sparsely connected with no self loops. An initially random network is optimised with the Stability-Optimised Circuit (SOC) algorithm [8] with *I*/*E* = 40 (see Methods). The additional outliers are calculated by linearly scaling all inhibitory weights to *I*/*E* = 3, 5, 10, 20, 40.

The existence of the negative outlier together with the zero trace condition has an interesting effect in upper triangular matrices. The larger the value of the outlier (in absolute value), the bigger the amplification (Fig 6A) and the number of amplified directions (Fig 6B). On one hand, this can be explained by the fact that more real parts are pushed to the right, creating longer decay envelopes, hence prolonging the time for the hidden feedforward structure to be amplified. On the other hand, this is not the sole source of the increased amplification; the combination of a large negative outlier with the zero trace condition has an additional non-intuitive effect on the geometry of the eigenvectors, i.e., it gives rise to larger eigenvector overlaps (Fig 6C). Without the zero trace condition, increasing the (negative) value of the outlier does not give rise to the same levels of amplification (S7 Fig), confirming that large amplification is an effect from the combination of the negative outlier and the zero trace condition. Interestingly, the negative outlier, *γ*, reflects the level of global inhibition [2, 8, 12, 29], such that (see Methods)

$$\begin{array}{c} \gamma \approx E[1-(\frac{I}{E})], \end{array}$$ (7)

where *E* and *I* are the sum of excitatory and inhibitory weights per postsynaptic neuron, respectively (Fig 6D).

#### The eigenspectrum norm is influenced by global inhibition

Taking into account that a large outlier reflects a connectivity matrix with inhibition dominating over excitation [12], i.e., the mean of inhibitory weights is larger than the mean of excitatory weights (Fig 6D), we tested whether strengthening the inhibitory weights would have a similar effect as increasing the (absolute) value of the outlier. For that, we built networks with excitatory and inhibitory neurons with an initial spectral radius *R*_outer_ = 10 and various ratios of inhibition and excitation, $\left(\frac{I}{E}\right)$. Non-zero excitatory and inhibitory weights are defined as $w_{0}/\sqrt{N}$ and $-(\frac{I}{E})w_{0}/\sqrt{N}$, respectively, with

$$\begin{array}{c} w_{0}=\frac{R_{\text{outer}}}{\sqrt{\frac{p(1-p)}{2}[1+(\frac{I}{E})]}}, \end{array}$$ (8)

where *p* is the probability of a connection being non-zero, taken from a uniform distribution. As a result, the eigenspectrum is mostly distributed inside a circle in the complex plane of radius *R*_outer_ [2] with one negative outlier due to inhibition dominating over excitation [12], given by (see Methods)

$$\begin{array}{c} \gamma \approx \frac{R_{\text{outer}}p[1-(\frac{I}{E})]}{\sqrt{\frac{2p(1-p)}{N}[1+(\frac{I}{E}{)}^{2}]}}. \end{array}$$ (9)

Importantly, an inhibition dominated network is characterised by a non-uniform distribution of eigenvalues inside the circle of radius *R*_outer_, with a denser region near the origin. The denser region is limited by an inner circle [2] with radius given by (see Methods)

$$\begin{array}{c} R_{\text{inner}}\approx \frac{R_{\text{outer}}}{\frac{1}{2}[1+(\frac{I}{E}{)}^{2}]}. \end{array}$$ (10)

**Fig 7:**
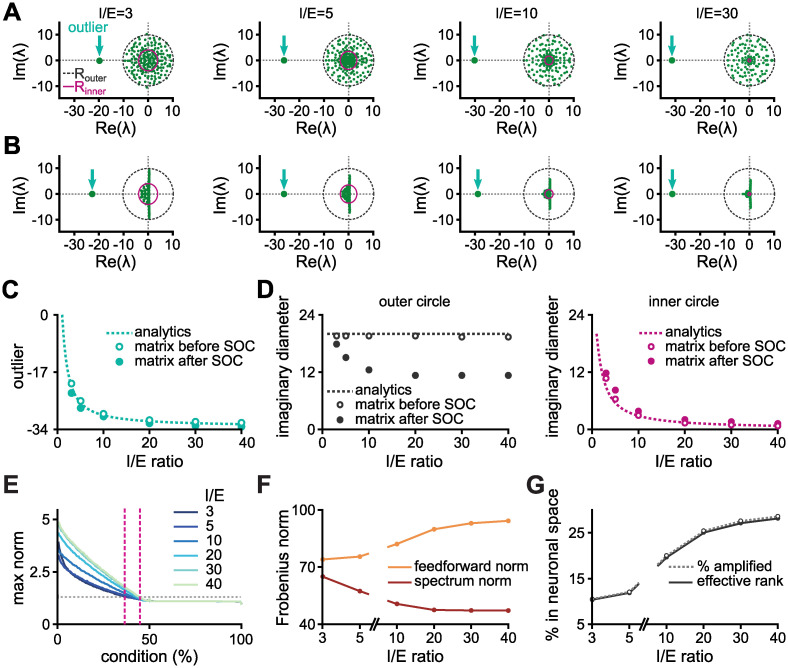
The effect of inhibitory dominance in Dalean matrices. **A**, Eigenspectra of connectivity matrices satisfying Dale’s law: 100 excitatory and 100 inhibitory neurons sparsely connected (probability of connection, *p* = 0.1) without self loops, constructed with spectrum of radius 10 and global inhibitory dominance of strength *I*/*E* (indicated on top of each panel). Outlier, outer radius (*R*_outer_), and inner circle (*R*_inner_) are highlighted. See Methods for details. **B**, Eigenspectra of connectivity matrices from panel A after optimising inhibitory weights with the SOC algorithm (see Methods for details). **C**, Value of the purely real outlier before (open circle) and after (closed circle) optimisation. Dashed line represents the analytical expression (Eq 9). Circles correspond to average over 1000 realisations. **D**, Imaginary diameter of the outer (left) and inner (right) circles. Open and closed circles represent average values before and after SOC optimisation algorithm, respectively, for 1000 random realisations. The outer and inner radii are calculated as the radius for which the density of imaginary elements drops below 0.005 and below half the maximum density, respectively. Dashed grey line (left) indicates *d*_im_ = 20, and purple dashed line (right) represents the analytical expression (Eq 10). **E**, Maximum norm per initial condition for different *I*/*E* ratios. Grey dotted line corresponds to a response norm that is 50% larger than the norm of the initial condition. Pink dashed lines indicate the percentage of initial conditions that elicit transients with maximum norm larger than 50% for *I*/*E* = 3 (lower percentage) and *I*/*E* = 40 (higher percentage). We linearly scale all weights to keep the same Frobenius norm (equal to 100) for comparison. **F**, The spectrum and the feedforward norms for different values of *I*/*E* in the corresponding real Schur transformation. **G**, Percentage of amplified conditions and effective rank of the corresponding matrix P (defined in the text) in the linear case.

Thus, the eigenspectrum of a inhibition dominated network is characterised by the presence of a negative outlier and a denser region of eigenvalues near the origin (Fig 7A); properties that are preserved after stabilisation of the weight matrix via the SOC algorithm (Fig 7B). The outlier’s value is not drastically changed by the SOC algorithm and is well captured by the analytical expression from Eq 9 (Fig 7C). Considering only the imaginary distribution, the outer circle shrinks while the inner circle remains mostly constant (Fig 7D), the latter being well described by Eq 10.

The existence of a denser region of imaginary elements near the origin suggests an effect similar to shrinking the imaginary distribution. This effect may be augmented by the lack of autapses, which moves the real elements towards the stability maximum to accommodate for the large negative outlier that emerges from inhibition dominating over excitation. We find that, indeed, larger global inhibitory strength leads to more amplified conditions and also to slightly larger amplification per condition when the Frobenius norm of the weight matrix is kept fixed (Fig 7E). By assigning larger values to the inhibitory weights, the spectrum norm decreases and the feedforward norm increases (Fig 7F). To highlight this finding, we note that when the inhibitory to excitatory ratio is large, *I*/*E* = 40, the strength of every nonzero excitatory-to-excitatory connection is 0.08, and yet the network is capable of stronger amplification compared to when *I*/*E* = 3 in which the nonzero excitatory-to-excitatory weights are set to 1.05 (see Eq 8).

Finally, the new amplified conditions induced by the strongest inhibition do not share their first principal component directions in their dynamical responses (Fig 7C), i.e., the noise robustness of the system is not compromised in this case [6]. This is possible because we are still in the short transient regime; the long transient regime cannot be reached by solely increasing the global inhibitory strength, i.e., large feedforward norms are always accompanied by small spectrum norms in stability-optimised circuits (S8 Fig), which restricts the accessibility to the different dynamics regimes of transient amplification (S6(D)–S6(F) Fig). Since the overall norm of the matrix stays the same (for comparison reasons), further increasing the inhibitory dominance would unavoidably decrease the excitatory weights even further. Therefore, the amplification power of the network through this mechanism eventually saturates before reaching the long transient regime. Notice that the Frobenius norm of the weight matrix was constrained by our choice of the initial spectral radius, *R*_outer_, that, consequently, constrains the possible spectrum and feedforward norms.

**Fig 8:**
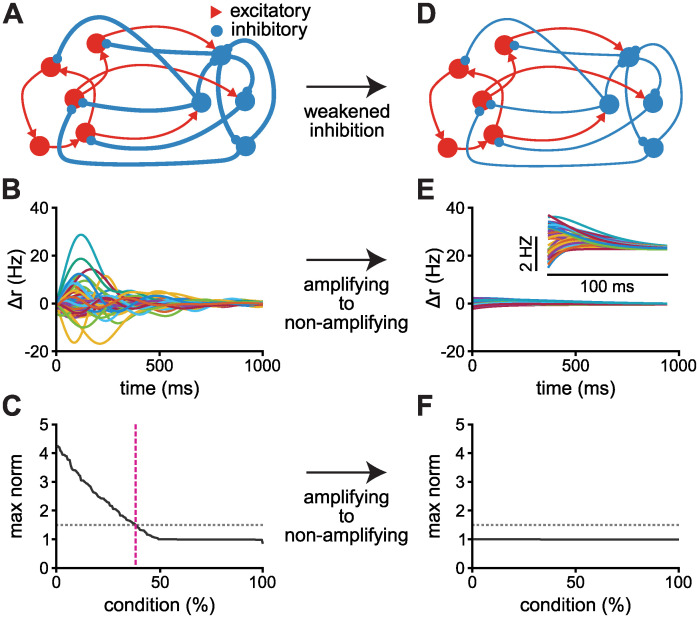
From short transient to non-amplifying with weakened inhibition. **A**, Schematic of a network with strong excitatory and inhibitory connections and *I*/*E* ratio of 40. **B**, The network’s dynamics given the preferred initialisation. The resulting network is in the short transient regime; the preferred initialisation yields amplifying dynamics. **C**, Maximum response norm for all orthogonal conditions, in decreasing order. Grey dotted line corresponds to a response norm that is 50% larger than the norm of the initial condition. Pink dashed line indicates the percentage of initial conditions with maximum norm larger than 50% of the initial condition. **D**, Schematic of the same network from panel A, but the inhibitory weights are scaled down by a factor of 40 (yielding an *I*/*E* ratio of 1), which could be interpreted as the resulting effect of modulation of the inhibitory neurons (or synapses). **E**, The dynamical response given the preferred initialisation; inset depicts the same dynamics on a different scale. **F**, Maximum response norm per condition. The network is unable to amplify any inputs. The maximum norm of the dynamics is equal to the norm of the initial condition (set to be 1) for all initialisations. The network is composed by 100 excitatory and 100 inhibitory neurons sparsely connected and without self loops. The schematics (panels A and D) is adapted from ref. [8].

These results indicate that the strength of the global inhibition, if modulated by an external signal, could thus serve as a dynamical switch between amplifying and non-amplifying regimes, and counter-intuitively, weakening of inhibitory synaptic weights would decrease the level of amplification of an originally amplifying network. To test this hypothesis, we built a network with strong inhibitory connections (*I*/*E* = 40) that enables the network to amplify certain inputs in the short transient regime (Fig 8A–8C). This network transitions into a network that is unable to amplify any inputs by scaling its inhibitory weights down (Fig 8D–8F), confirming that global inhibition can indeed serve as an external switch that controls whether the network is able or not to transiently amplify inputs.

#### The diameter of the imaginary distribution is influenced by the relationship between excitatory-to-inhibitory and excitatory-to-excitatory connections

**Fig 9:**
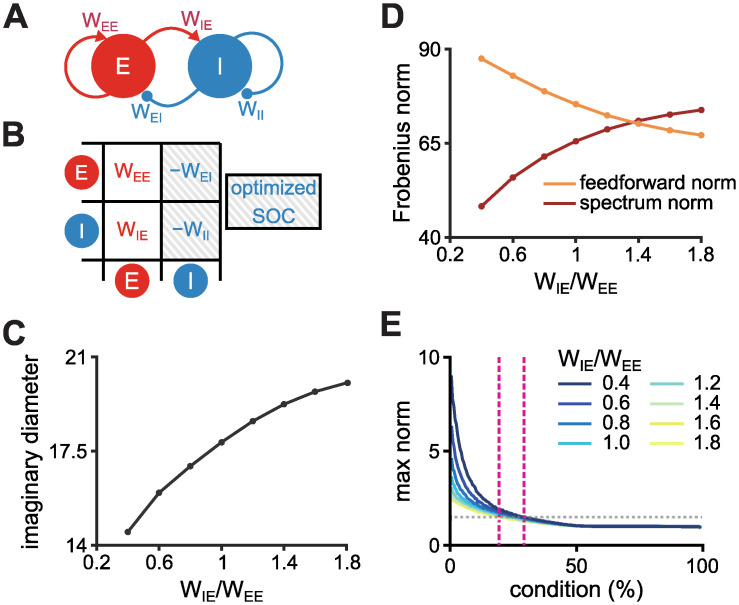
The relationship between E-to-I and E-to-E connectivity strengths alters the imaginary distribution of eigenspectra of Dalean networks. **A**, Schematics of the mean-field analysis of a network with a group of excitatory (E) and a group of inhibitory (I) neurons. The mean weight from E-to-E, E-to-I, I-to-E, and I-to-I are represented by *W*_EE_, *W*_IE_, *W*_EI_, and *W*_II_, respectively. **B**, Weight matrix of a simplified network from panel A [10]. Inhibitory connections are optimised by the SOC algorithm [8]. **C**, Imaginary diameter of a network with 100 excitatory and 100 inhibitory neurons as a function of the ratio E-to-I to E-to-E weights. **D**, Feedforward (orange) and spectrum (red) norm as a function of the ratio E-to-I to E-to-E weights for the same networks from panel C. **E**, Maximum norm per initial condition for different ratios E-to-I to E-to-E weights. Grey dotted line indicates response norm that is 50% larger than the norm of the initial condition. Pink dashed lines indicate the maximum percentage of orthogonal initial conditions that evoke response norm 50% larger than initial condition for *W*_IE_/*W*_EE_ = 1.8 (lower percentage) and *W*_IE_/*W*_EE_ = 0.4 (higher percentage).

In the upper-triangular version of the weight matrix, $\tilde{\text{W}}$, the eigenspectrum’s imaginary distribution is given by the off-diagonal terms of $\tilde{\text{W}}$ (see Fig 1B). However, for a biologically plausible weight matrix, **W**^**B**^, the manipulation of the imaginary distribution is not as trivial [2, 12]. To get an intuition of the role of each connection type, we use a mean-field approach in which a large weight matrix is simplified as a 2-by-2 matrix [10]: rows and columns correspond to the excitatory and inhibitory populations (Fig 9A), and the elements represent their mean connections (Fig 9B). We define *W*_EI_, *W*_IE_, *W*_EE_, and *W*_II_ (all positive) as the mean connectivity strength of inhibitory-to-excitatory (I-to-E), excitatory-to-inhibitory (E-to-I), excitatory-to-excitatory (E-to-E), and inhibitory-to-inhibitory (I-to-I) groups, respectively. The eigenvalues are complex when the condition 4*W*_EI_*W*_IE_ > (*W*_EE_ + *W*_II_)^2^ is satisfied (see Methods for detailed calculation). Real and imaginary parts of the two eigenvalues corresponding to the simplified matrix are thus

$$\begin{array}{c} \text{Re}(\lambda_{1})=\text{Re}(\lambda_{2})=\frac{1}{2}(W_{\text{EE}}-W_{\text{II}})\;\text{and} \end{array}$$ (11)

$$\begin{array}{c} \text{Im}(\lambda_{1,2})=\pm \frac{1}{2}\sqrt{4W_{\text{EI}}W_{\text{IE}}-(W_{\text{EE}}+W_{\text{II}}{)}^{2}}. \end{array}$$ (12)

When inhibitory connections, *W*_EI_ and *W*_II_, are optimised for stability [8], we are left with excitatory connections to manipulate the imaginary diameter of the eigenspectrum. Weakening E-to-I, *W*_IE_, or strengthening E-to-E, *W*_EE_, should shrink the imaginary distribution according to Eq 12.

To test the intuition from the mean-field analysis, we built networks with connectivity matrices obeying Dale’s law and without self loops according to the Stability-Optimised Circuit (SOC) algorithm [8] with varying ratios *W*_IE_/*W*_EE_. For each network initialisation, we systematically increased the strength of E-to-I connections, *W*_IE_, while keeping the same strength of E-to-E connections, *W*_EE_. We then optimised inhibitory connections (both *W*_EI_ and *W*_II_) with the SOC algorithm, and linearly scaled all weights to maintain the same Frobenius norm of the connectivity matrix, ‖**W**^**B**^‖, for all realisations. We found that, in line with Eq 12, the imaginary diameter of the Dalean weight matrix enlarges with the increase of the ratio *W*_IE_/*W*_EE_ (Fig 9C). As a consequence, the spectrum norm increases as the feedforward norm decreases with the strengthening of the E-to-I compared to E-to-E connections (Fig 9D). This indicates that in a stabilised network with connectivity following Dale’s law, i.e., when inhibitory connections are optimised for stability, changing the relationship between E-to-I and E-to-E connectivity can influence the diameter of the imaginary distribution of eigenvalues. Due to a decrease in the imaginary diameter, the max norm of dynamics evoked by the orthogonal initial conditions are slightly larger when E-to-I are weaker than E-to-E weights (Fig 9E). These results indicate that excitatory-to-inhibitory connections may play a role in shaping the network’s dynamics through their effect on the imaginary distribution of the connectivity matrix.

## Discussion

In this article we used upper triangular matrices as abstract representations of the dynamical properties of a connectivity matrix to control the quantities that are relevant for the neural dynamics in the transient amplification regime. By by-passing, temporarily, the connectivity matrix and focusing on a hypothetical Schur transformation, we found new dynamical regimes of large amplification that translated into long transients in non-linear networks. We showed that the amount of transient amplification that a network can produce may be controlled by the ratio between the norms of the spectrum and hidden feedforward structure. Increasing the feedforward norm or decreasing the eigenspectrum’s imaginary diameter resulted in larger amplifying dynamics.

Different combinations of the eigenspectrum’s and feedforward norms resulted in regimes of transient amplification that lay in subspaces with different dimensionalities and evolved with distinct timescales for non-linear networks. Very short transient periods in non-linear networks spanned the entire output space but only a few orthogonal conditions elicited large amplification. Short transient periods in non-linear networks lay in a lower subspace, but more orthogonal conditions elicited large amplification. Finally, long transient periods in non-linear networks lay in the same low-dimensional subspace, but many orthogonal initial conditions evoked large amplification. The real distribution could considerably change the levels of amplification, but did not affect the relationship between amplification and spectrum or feedforward norms. When the majority of real parts were close to the upper bound of stability, a zero trace condition had an interesting effect: the larger the negative outlier, the closer the real parts were to the stability limit, thus creating larger amplification levels. Moreover, we found a trade-off between the number of orthogonal initial conditions that elicit large amplifications (larger than 50% of initial condition) and the dimensionality of the underlying neuronal dynamics. The source of amplification, i.e., the overlaps of the eigenvectors, inevitably restricted the subspace in which the dynamical outputs evolve, indicating that any noise added to the system is also amplified in the same subspace, giving rise to low robustness to noise [6].

In biologically plausible networks that satisfy Dale’s law (i.e., with excitatory and inhibitory neurons), we found that stronger global inhibitory dominance controlled the spectrum norm due to its relationship with the eigenspectrum outlier and a non-uniform distribution of the remaining eigenvalues. Additionally to global inhibition affecting the spectrum norm, we also showed that excitatory-to-inhibitory connections, more specifically its relationship to excitatory-to-excitatory connections, can alter the eigenspectrum’s imaginary diameter. We could therefore link the results from upper triangular matrices and biologically plausible networks via two properties related to specific connectivity patterns: global inhibition and excitatory-to-inhibitory connectivity.

Our work complements previous studies relating manipulations of eigenspectrum and their consequence to neuronal dynamics (e.g., [2, 6, 8, 10, 12, 14, 21, 30]), more specifically on the topic of transient amplification (e.g., [6, 8, 10, 12, 21]). Transient amplification is a phenomenon tightly linked to eigenspectrum and non-normality of a connectivity matrix. For the dynamics to be transient, the network’s activity must eventually decay back to baseline, which constrains the eigenspectrum’s real part to be less than unity—a stability constraint. For the dynamics to amplify certain inputs, the connectivity matrix must be non-normal [6, 8, 10]. Neuronal networks with excitatory and inhibitory neurons are always non-normal, because of the separation of positive and negative columns [10], but non-normality of a connectivity matrix is not sufficient for a network to transiently amplify inputs [6]. A necessary condition in, e.g., random matrices is that the symmetric (real) part of the connectivity matrix has eigenvalues greater than unity [6]. In networks with sparsely connected excitatory and inhibitory neurons, amplification levels increase with the eigenspectrum radius and the connectivity sparsity, which reflects the global strength of excitatory and inhibitory connections [12]. If the radius is greater than the unity, the network’s dynamics are unstable, which can be stabilised by iteratively adjusting inhibitory connections to decrease the spectral abscissa until its values is less than one (and therefore stable) [8]. This algorithm, referred to as Stability-Optimised Circuits (SOC), and used here to generate Dalean matrices, is able to optimise networks to elicit largely amplified transients. Interestingly, after optimisation, the eigenspectrum retains a similar imaginary diameter but the eigenspectrum’s real part accumulates near the stability limit apart from the outlier [8]. We confirmed that in Dalean networks, the existence of a large negative outlier imposes that the remaining of the eigenspectrum’s real elements be pushed near the spectral abscissa in networks without autapses.

Any structure imposed to a connectivity matrix affects its eigenspectrum in a particular way. Excitatory-inhibitory balance constrains the eigenspectrum to a well defined circle for finite-sized networks, with a non-uniform distribution of eigenvalues inside the circle when the distribution of excitatory and inhibitory weights differ [2]. Cyclic connectivity is reflected by eigenvalues lying on a circle (as oppose to inside for random matrices) [14], and the combination of random matrix and a single feedforward chain with purely imaginary eigenvalues of alternating sign results in an eigenspectrum with two centres at ±*i*, results in large amplification of oscillatory inputs [21]. Complementary to these previous studies, our work explored simple changes in the eigenspectrum of a connectivity matrix, such as the diameter of imaginary distribution, real negative outlier, and zero trace condition. More importantly, we could directly link these simple changes to properties of Dalean matrices, shining a light on how such changes might be implemented in biologically plausible networks.

Our results in matrices following Dale’s law allowed us to find a potential role for changes in global inhibition in neuronal networks. Quick changes in the efficacy of inhibitory synapses, both inhibitory-to-inhibitory and inhibitory-to-excitatory, could act as a switch in the dynamical state of the network, changing from amplifying to non amplifying by reducing all inhibitory efficacies. This switch may be controlled by neuromodulators such as the neuropeptide proctolin [31], acetylcholine [32], dopamine [33], or serotonin [34], as well as stereotypical connectivity motifs [35, 36] that could influence inhibitory activity in a similar way to weakening or strengthening all inhibitory weights. A modulatory increase in the global levels of inhibition could be thus allowing the system to amplify inputs, facilitating signal processing in the brain. This effect resembles the *paradoxical* decrease in inhibitory activity as a result of an increase of external (excitatory) input onto inhibitory neurons [37]. We did not implement any external input, and thus the counter-intuitive (or paradoxical) effect originates from scaling all inhibitory weights up or down. Intuitively, one would expect that by increasing inhibitory weights, the amplitude of the network activity should decrease, and vice-versa. However, we counter-intuitively found the opposite to be true.

The dynamics of real biological neuronal networks, assessed from large-scale recordings of neural activity, has been shown to typically lie in a low-dimensional space [38], i.e., the number of dimensions necessary to explain the majority of the data variance is much smaller than the number of neurons recorded. The low-dimensional dynamics seen in experiments are well described by, e.g., non-liner rate networks (such as the one used in our work), when connections are optimised for either the experiment’s task [7] or recorded neuronal activity [39]. Complementary to large-scale recordings of neuronal dynamics, recent technological improvements have allowed researchers to build connectivity maps of large-scale brain regions [40]. Finding the relationship between connectivity and dynamics is thus crucial to understand the function of brain circuitry. The rank of the weight matrix carries information about the dimensionality of the underlying dynamics generated by the network, but it does not necessarily indicate the dimensionality of the subspace in which the dynamics lie. When, e.g., a weight matrix is constructed as a sum of a unit-rank (generated from two independent vectors) and a random (zero mean) matrix, the underlying dynamics evolves along the direction of the vector used to generate the unit-rank matrix [22]. These vectors are the eigenvectors of the unit-rank matrix [22], and because of the construction of the weight matrix, both weight and eigenvector matrices will share the same effective rank. We have shown a more general case in which the effective rank of the eigenvector matrix can carry information about the dimensionality of the dynamics, even when the weight matrix itself has a high effective rank.

We limited our exploration in upper triangular matrices to the effects of simple changes in the spectrum and feedforward structures, by controlling their range and norm. In networks that satisfy Dale’s law, excitatory weights were sparsely connected with the same connection strength for all non-zero entries, changing only the ratio between excitatory-to-inhibitory (E-to-I) and excitatory-to-excitatory (E-to-E) connections. In reality, synapses are constantly changing, following stereotypical synaptic plasticity rules that allow learning of memories [5, 41–43] or additional types of computation such as dynamical switch [44] and input categorisation [45]. Most computational models typically explore learning as a process involving changes in excitatory-to-excitatory (E-to-E) connections accompanied by modifications in inhibitory-to-excitatory (I-to-E) connections for stability [41, 42]. Typically, excitatory-to-inhibitory (E-to-I) connections are randomly initialised and kept fixed throughout the simulations (but see ref. [46]). Our results linking E-to-I connections to the eigenspectrum’s imaginary distribution, and consequently, amplification levels, suggest a role for such connection type. Future theoretical and experimental work would be thus necessary to design and measure potential plasticity rules for E-to-I connections that, in synergy with E-to-E [47] and I-to-E plasticity [44], may, e.g., generate new classes of activity patterns.

Our work opens the door for the exploration of new questions related to neuronal dynamics, such as how the structure—besides the norm—of the feedforward part as well as how non-uniform imaginary distributions affect the dynamics of biologically plausible networks.

## Methods

### Details for upper triangular matrix setup

To construct the upper triangular matrices we take inspiration from the real Schur transformation of real matrices. In this form, the matrix is upper triangular with some 2 × 2 blocks on the diagonal. These blocks have real entries and their eigenvalues are the complex eigenvalues of the initial matrix (a pair of conjugates). We fix the triangular part that is not involved in the eigenvalue blocks (more details on this below) and assign to it the norm that we wish by scaling its entries. For the manipulation of the spectrum we construct our distributions as follows. To have the pair of complex eigenvalues *α* ± *βi* in the spectrum, we add the block

$$\begin{array}{c} \left(\begin{array}{cc} \alpha & -\beta \\ \beta & \alpha \end{array}\right) \end{array}$$

along the diagonal. For real eigenvalues, we add the corresponding real value on the diagonal. The resulting matrix is as follows (example of a 5-by-5 upper triangular matrix with 4 complex eigenvalues and 1 real eigenvalue):

$$\begin{array}{c} \tilde{\text{W}}=(\begin{array}{ccccc} \alpha_{1} & -\beta_{1} & \phi_{1} & \phi_{2} & \phi_{3}\\ \beta_{1} & \alpha_{1} & \phi_{4} & \phi_{5} & \phi_{6}\\ 0 & 0 & \alpha_{2} & -\beta_{2} & \phi_{7}\\ 0 & 0 & \beta_{2} & \alpha_{2} & \phi_{8}\\ 0 & 0 & 0 & 0 & \gamma \end{array}) \end{array}$$ (13)

The eigenvalues are λ_1,2_ = *α*_1_ ± *iβ*_1_, λ_3,4_ = *α*_2_ ± *iβ*_2_, and λ_5_ = *γ*, while the feedforward structure is defined by {*ϕ*_*i*_}, *i* = 1, …, 8. Note that all elements of $\tilde{\text{W}}$ are purely real. For consistency, we chose to keep the percentage of purely real eigenvalues to 3%, i.e., 6 out of the 200 eigenvalues are purely real, and 194 are complex.

#### Real distributions

The real distributions we use in this work are the following:

- **A single valued distribution**: all real parts are the same and equal to a fixed value. We use Re(λ_*k*_) = 0, ∀*k* in Fig 3A (light pink) and Fig 3B, as well as S2 Fig (pink) and S6(B) and S6(E) Fig; Re(λ_*k*_) = −0.5, ∀*k* in Fig 3A (dark pink) and S2 Fig (dark pink); and Re(λ_*k*_) = 0.5, ∀*k* in S2(A) Fig (light pink).
- **A distribution with a negative outlier**: in this construction we add a purely real negative outlier, λ_out_, at a specific point in the complex plane. In Fig 6A–6C and S6(C) Fig, a number *M* = λ_out_/*α*_max_ = 2λ_out_ of eigenvalues are equal to *α*_max_ = 0.5 and the rest is equal to zero so that the zero trace condition is satisfied. The value of the outlier is indicated in the figure legends. In S7 Fig the real part of all other eigenvalues is equal to zero, and thus the zero trace condition is not satisfied.
- **A uniform distribution on the interval (−0.5, 0.5)**: all real parts, except for the last one, are distributed uniformly between the values −0.5 and 0.5. As before, because of the zero trace condition, we have to add a small outlier (the last real eigenvalue) to complement for the non-zero sum of the rest of the values. We use this type of distribution in Figs 3A (light green), 4 and 5, as well as S1, S3, S4, S5, S6(A), S6(D) and S6(F) and S9 Figs.
- **A uniform distribution on the interval (0.5 − d_re_, 0.5)**: all real parts are distributed uniformly between the values 0.5 − *d*_re_ and 0.5, creating a uniform distribution with diameter *d*_re_ and a maximum value of 0.5. We use this type of distribution in Fig 3A (dark green) with *d*_re_ = 10 and S2(C) Fig with *d*_re_ = {0, 0.1, 1, 10, 100}.

In all cases, the pairings of the real parts with the corresponding imaginary parts are random—except for forcing the conjugacy of eigenvalues, that is, we make sure that the same real part is paired with conjugate imaginary parts. All simulations are run for 200 realisations (except where noted), with respect to the randomness of the imaginary distribution, and final quantities are averaged across all realisations for plotting.

#### Feedforward structures

In the simulations shown in Fig 2, S2(A) and S2(B) Fig (pink), and S6(D)–S6(F) Fig, the feedforward structure of the upper triangular matrix is taken to be equal to the upper triangular part of the Schur transform of a fixed Stability-Optimised Circuit (SOC) [8] with *I*/*E* = 3 (explained below). The SOC is a matrix known to create strong non-normal amplification and its corresponding feedforward structure is not random, but finely tuned to create amplification. In the simulations shown in Figs 3–6, as well as S1, S2(B) (yellow), S3, S4, S5, S6(A)–S6(C), S7 and S9 Figs, the upper triangular part of the matrix is drawn from a uniform distribution on the interval (−0.5, 0.5) and scaled accordingly to match a specific Frobenius norm.

#### Imaginary clustering at different points

To understand whether the surprising effect that arises from shrinking the imaginary spectrum is due to the clustering of the eigenvalues, we checked what happens when the imaginary parts of the eigenvalues are not uniformly distributed around zero, but clustered around symmetrically displaced points on the imaginary axis (S1(A) Fig inset). In this case the linear responses exhibit an interesting phenomenon, resembling the beats in acoustics (S1(A) Fig). Because the frequencies are close to each other (due to the clustering), the amplitudes of the different neuronal responses are superimposed when phased, creating a response of very high amplitude (which by our definition would count as amplification). Moreover, the differences in the frequencies create an envelope that is modulating this amplitude over time. The nonlinear responses fail to capture most of the interesting dynamics seen linearly and do not amplify to the same extent (S1(B) Fig). The very high frequency makes it impossible for any potentially amplifying mode to drive the rest of the modes and create a large amplified response. Because of this discrepancy between linear and nonlinear behaviour, we do not consider these regimes as amplifying for the purposes of this manuscript. It is worth noting that similar behaviour to the ±100 example is seen when clustering the imaginary spectrum at different nonzero values (S1(C) Fig).

### Eigenvector overlaps

Recall that the eigenvectors are, in general, complex, in conjugate pairs and that in order to compute the overlap between the eigenvectors we need to consider their inner product. The inner product of two complex vectors is defined as

$$\begin{array}{c} \left\langle a,b\right\rangle =\sum_{i}a_{i}\bar{b_{i}} \end{array}$$ (14)

and the angle, *θ*, between two complex vectors is given by

$$\begin{array}{c} \text{cos}\left(\theta \right)=\frac{\text{Re}(\langle a,b\rangle )}{\left\|a\|\|b\right\|} \end{array}$$ (15)

Therefore, to compute the angles between the eigenvectors we use Eq 15. In particular, we normalise the eigenvectors to unit norm and compute all pairwise angles. Finally, since cos(*π* − *θ*) = −cos(*θ*), when computing the percentage of small eigenvector overlaps (i.e., less than 45°), we consider as angle the minimum angle between *θ* and *π* − *θ*. We would like to note here that non-normality depends on the complex inner product between eigenvectors, and not only its real part. However, we have chosen to compute this more intuitive version of an angle between two complex vectors (which is commonly used in the literature) as a characterisation of the amplification dynamics. We compare these results with an alternative computation of the eigenvector overlap in S9 Fig.

### Dimensionality of dynamics—Effective rank of eigenvector matrix

Here we briefly explain the intuition behind the effective rank of the eigenvector matrix **V**. This is understood as the number of significant dimensions in the range of a matrix. For example, if the effective rank is equal to *κ*, then a trajectory evoked by a random initial condition in the range of **V** is sufficiently approximated by *κ* dimensions (see section 3 of ref. [24]). The fact that the effective rank of the eigenvector matrix is small indicates that there are a few prevalent directions in the space spanned by the eigenvectors, which indicates that dynamical trajectories will be biased towards a small subspace of the entire eigenvector space. This is further explored and verified with the computation of the dynamical matrix P defined below.

#### Construction of the matrix P

We construct the matrix P to understand how correlated the dynamics of the network are, given different initial conditions. This matrix represents the prevalent directions of the dynamics, given different initialisations. This is done as follows: after having identified the optimal orthogonal basis of initial conditions $I_{B}$, we initialise the network at each of the vectors in this basis, one at a time. For each such vector, if the induced dynamics are amplified, i.e., if the norm of the rate vector is at some point in time larger than 1.5 (the initialisation vectors have always unit norm), then we perform Principal Component Analysis on the dynamics. More specifically, we compute the eigenvectors of the covariance matrix of the neuronal dynamics for each of these simulations. We only consider the eigenvector corresponding to the largest eigenvalue and store it as a column in the matrix P. Once we have initialised the network at all vectors in $I_{B}$ we are left with a *N* × *M* matrix P. The number *M* is the same as the number of conditions that lead to an amplified response and provides a maximum bound for the effective rank of matrix P.

The effective rank of P thus gives us the effective dimensionality of the space spanned by the columns of P. If the effective rank is less than the number of columns, we can deduce that orthogonal initial conditions have first principal components that are closely aligned to each other in state-space. This implies that the initial network amplifies orthogonal initial conditions along the same low dimensional subspace.

In S3 Fig we also compare the effective rank of the matrix P when the number of principal components stored as columns (for each amplified initialisation) is such that the total variance captured is greater than 85%. In that case, the matrix P has size N×K. Here, $K=\sum_{i=1}^{M}\kappa_{i}$, where *κ*_*i*_ is the number of principal components needed to explain at least 85% of the variance of the neuronal response, when initialised at the *i^th^* (amplified) condition of $I_{B}$. We find similar results, i.e., in the long transient regime there is a big discrepancy between the total number of columns (K) and the effective rank of the dynamical matrix P. It is worth mentioning that the effective rank is bounded by the number of neurons *N*. The fact that even though the number of columns is much larger than *N* in the long transient, but the effective rank still fails to reach its bound, verifies the intuition obtained by the main results in Fig 4.

### Construction of recurrent networks satisfying Dale’s law

The recurrent networks satisfying Dale’s law are constructed following the Stability-Optimised Circuit (SOC) algorithm [8]. In our simulations, 50% of the neurons are excitatory and 50% inhibitory, so that the first half columns of **W**^**B**^ are strictly positive and the second half strictly negative. The connections are initially generated at random from a uniform distribution with probability of connection *p* = 0.1 and individual weights given by: $w_{\text{E}}=w_{0}/\sqrt{N}$ for excitatory and $w_{\text{I}}=-(\frac{I}{E})w_{0}/\sqrt{N}$ for inhibitory, where $\left(\frac{I}{E}\right)$ is the ratio between inhibition and excitation, and *N* is the size of the network. The initial weight, *w*_0_, is defined as

$$\begin{array}{c} w_{0}=\frac{R_{\text{outer}}}{\sqrt{\frac{p(1-p)}{2}[1+(\frac{I}{E}{)}^{2}]}}, \end{array}$$ (16)

where *R*_outer_ = 10 is the radius of the eigenspectrum distribution. After the initialisation of a matrix, we implement the SOC optimisation algorithm, which modifies only inhibitory weights to enforce that the spectral abscissa is less than 0.5 (max_λ_Re(λ) = *α*_max_ = 0.5). In this process, both zero and non-zero inhibitory connections are modified. Thus, to maintain a certain level of sparsity in inhibitory connections, we keep the density of inhibitory connections lower than 0.4. Additionally, we keep the absence of self loops and the same *I*/*E* ratio by linearly scaling all non-zero inhibitory weights. We impose different conditions to connections depending on which aspect of the connectivity we explore, as define below.

#### Varying *I*/*E* ratios

In Fig 7 and S8 Fig we pre-define the ratio *I*/*E* and then optimise the inhibitory connections. In Figs 6D and 8 we optimise the connectivity for *I*/*E* = 40. We linearly scale all inhibitory weights and calculate the resulting outlier in Fig 6D for *I*/*E* = 30, 20, 10, 5, 3. We linearly scale all inhibitory weights by the fraction 1/40 to get a resulting weight matrix with *I*/*E* = 1 in Fig 8.

#### Varying the E-to-I and E-to-E connections

In Fig 9 we implement the SOC algorithm with different combinations of the E-to-I and E-to-E connectivity strengths. We define our “standard” network, i.e., with the same E-to-I and E-to-E connectivity strengths (*W*_IE_/*W*_EE_ = 1) as described above, optimised using *I*/*E* = 3. For each ratio E-to-I to E-to-E, *W*_IE_/*W*_EE_ = {0.4, 0.6, 0.8, 1.2, 1.4, 1.6, 1.8}, we start with a new matrix with weights defined as above and then linearly scale all E-to-I non-zero weights by *W*_IE_/*W*_EE_. After scaling E-to-I weights, we run the SOC algorithm, as described above, keeping *I*/*E* = 3. Finally, after optimisation we linearly scale all weights so that the Frobenius norm of the resulting **W**^**B**^ is the same as the standard weight matrix, which has *W*_IE_/*W*_EE_ = 1.

### Analysis of a 3-by-3 upper triangular weight matrix

To get an intuition for the role of the eigenspectrum, feedforward norm, and outlier, we analyse a simplified 3-by-3 upper triangular matrix,

$$\begin{array}{c} \tilde{\text{W}}=(\begin{array}{ccc} \alpha & \beta & \phi_{2}\\ -\beta & \alpha & \phi_{1}\\ 0 & 0 & \gamma \end{array}). \end{array}$$ (17)

This matrix has three eigenvalues, λ_*k*_, given by

$$\begin{array}{c} \left\{ \begin{array}{c} \lambda_{1}=\gamma \\ \lambda_{2}=\alpha -i\beta \\ \lambda_{3}=\alpha +i\beta , \end{array}\right. \end{array}$$ (18)

where *α*, *β*, and *γ* can be interpreted as the average of the real part of the eigenspectrum distribution, the eigenspectrum’s imaginary diameter, and the outlier. The other two non-zero entries, *ϕ*_1_ and *ϕ*_2_, represent the feedforward structure with norm $\phi_{\text{norm}}=\sqrt{\phi_{1}^{2}+\phi_{2}^{2}}$.

The eigenvectors associated with λ_*k*_ are

$$\begin{array}{c} v_{1}=(\begin{array}{c} a_{1}\\ b_{1}\\ c_{1} \end{array})=(\begin{array}{c} \frac{-\alpha \phi_{2}+\gamma \phi_{2}+\beta \phi_{1}}{{\left(\alpha -\gamma \right)}^{2}+\beta^{2}}\\ \frac{-\alpha \phi_{1}+\gamma \phi_{1}-\beta \phi_{2}}{{\left(\alpha -\gamma \right)}^{2}+\beta^{2}}\\ 1 \end{array})c_{1}, \end{array}$$ (19)

$$\begin{array}{c} v_{2}=(\begin{array}{c} a_{2}\\ b_{2}\\ c_{2} \end{array})=(\begin{array}{c} i\\ 1\\ 0 \end{array})b_{2}, \end{array}$$ (20)

and

$$\begin{array}{c} v_{3}=(\begin{array}{c} a_{3}\\ b_{3}\\ c_{3} \end{array})=(\begin{array}{c} -i\\ 1\\ 0 \end{array})b_{3}. \end{array}$$ (21)

We can thus calculate the normalised inner product of the vectors,

$$\begin{array}{c} \frac{\left\langle v_{2},v_{3}\right\rangle }{\left\|v_{2}\|\|v_{3}\right\|}=0 \end{array}$$ (22)

and

$$\begin{array}{cc} \eta & \equiv \frac{\left\langle v_{1},v_{2}\right\rangle }{\left\|v_{1}\|\|v_{2}\right\|}=\frac{\bar{\left\langle v_{1},v_{3}\right\rangle }}{\left\|v_{1}\|\|v_{3}\right\|}\\ & =\frac{-[\phi_{1}\left(\alpha -\gamma \right)+\beta \phi_{2}]+i[\phi_{2}\left(\alpha -\gamma \right)-\beta \phi_{1}]}{\sqrt{2\{[\phi_{2}\left(\alpha -\gamma \right)-\beta \phi_{1}{]}^{2}+[\phi_{1}\left(\alpha -\gamma \right)+\beta \phi_{2}{]}^{2}+[{\left(\alpha -\gamma \right)}^{2}+\beta^{2}{]}^{2}\}}}\\ & =\frac{-[\phi_{1}\left(\alpha -\gamma \right)+\beta \phi_{2}]+i[\phi_{2}\left(\alpha -\gamma \right)-\beta \phi_{1}]}{\sqrt{2[\phi_{1}^{2}+\phi_{2}^{2}+(\alpha -\gamma {)}^{2}+\beta^{2}][{\left(\alpha -\gamma \right)}^{2}+\beta^{2}]}}. \end{array}$$ (23)

From Eq 23 we can calculate the absolute value of the normalised inner product of the non-orthogonal eigenvectors (Eq 4 in the main text),

$$\begin{array}{c} \left|\eta \right|=\sqrt{\eta \bar{\eta }}=\frac{\phi_{\text{norm}}}{\sqrt{2[\phi_{\text{norm}}^{2}+(\alpha -\gamma {)}^{2}+\beta^{2}]}}. \end{array}$$ (24)

We plot |*η*| (from Eq 24) as a function of *β* in Fig 3C and as a function of *ϕ*_norm_ in Fig 3D (values of *α* and *γ* are described in the caption). We also plot |*η*| (Eq 24) as a function of *β* and *ϕ*_norm_ representing the spectrum’s imaginary diameter and feedforward norm, respectively, in S6(G)–S6(I) Fig.

### Mean-field analysis of a 2-by-2 weight matrix with excitatory and inhibitory neuronal populations

To get an intuition for the role of the real and imaginary distributions of a Dalean weight matrix, we analyse a 2-by-2 matrix

$$\begin{array}{c} {\text{W}}^{\text{B}}=(\begin{array}{cc} W_{\text{EE}} & -W_{\text{EI}}\\ W_{\text{IE}} & -W_{\text{II}} \end{array}), \end{array}$$ (25)

where *W*_EE_, *W*_EI_, *W*_IE_, and *W*_II_ correspond to the mean excitatory-to-excitatory (E-to-E), inhibitory-to-excitatory (I-to-E), excitatory-to-inhibitory (E-to-I), and inhibitory-to-inhibitory (I-to-I) connections, respectively. The eigenvalues of this connectivity matrix are

$$\begin{array}{c} \left\{ \begin{array}{c} \lambda_{1}=\frac{1}{2}[W_{\text{EE}}-W_{\text{II}}+\sqrt{{\left(W_{\text{EE}}+W_{\text{II}}\right)}^{2}-4W_{\text{EI}}W_{\text{IE}}}]\\ \lambda_{2}=\frac{1}{2}[W_{\text{EE}}-W_{\text{II}}-\sqrt{{\left(W_{\text{EE}}+W_{\text{II}}\right)}^{2}-4W_{\text{EI}}W_{\text{IE}}}]. \end{array}\right. \end{array}$$ (26)

The eigenvalues are complex when

$$\begin{array}{c} 4W_{\text{EI}}W_{\text{IE}}>{\left(W_{\text{EE}}+W_{\text{II}}\right)}^{2}, \end{array}$$ (27)

with real part $\text{Re}\left(\lambda_{1}\right)=\text{Re}\left(\lambda_{2}\right)=\frac{1}{2}\left(W_{\text{EE}}-W_{\text{II}}\right)$ and imaginary part $\text{Im}\left(\lambda_{1}\right)=\frac{1}{2}\sqrt{4W_{\text{EI}}W_{\text{IE}}-{\left(W_{\text{EE}}+W_{\text{II}}\right)}^{2}}$ and $\text{Im}\left(\lambda_{2}\right)=-\frac{1}{2}\sqrt{4W_{\text{EI}}W_{\text{IE}}-{\left(W_{\text{EE}}+W_{\text{II}}\right)}^{2}}$. The imaginary part of both eigenvalues is small when the two quantities from Eq 27 are similar,

$$\begin{array}{c} 4W_{\text{EI}}W_{\text{IE}}⪆{\left(W_{\text{EE}}+W_{\text{II}}\right)}^{2}, \end{array}$$ (28)

and large when the inter-group connections are much greater than the intra-group connections,

$$\begin{array}{c} 4W_{\text{EI}}W_{\text{IE}}\gg {\left(W_{\text{EE}}+W_{\text{II}}\right)}^{2}. \end{array}$$ (29)

Therefore, in realistic Dalean networks with excitatory and inhibitory neurons, the imaginary diameter can be controlled by the inter-group connectivity strength, with small imaginary diameters by enforcing the relationship between mean weights according to Eq 28.

### Relationship between negative outlier and inhibition-to-excitation ratio

To find the relationship between the negative outlier and the inhibition-to-excitation ratio, *I*/*E*, we consider a special case in which the sum of all rows of a biological weight matrix, **W**^**B**^, are equal to *γ*,

$$\begin{array}{c} \sum_{j=1}^{N}w_{ij}^{\text{B}}=\gamma ,\forall i, \end{array}$$ (30)

where *N* is the number of neurons. Additionally, we consider that the sum of excitatory and inhibitory weights in each row is the same,

$$\begin{array}{c} \sum_{j=1}^{N/2}w_{ij}^{\text{B}}=E\text{,}\;\sum_{j=\frac{N}{2}+1}^{N}w_{ij}^{\text{B}}=-I\text{,}\;\text{and}\;E-I=\gamma ,\forall i. \end{array}$$ (31)

In this particular case the outlier is

$$\begin{array}{c} \gamma =E-I=E(1-\frac{I}{E}). \end{array}$$ (32)

We extrapolate this to a random weight matrix with structured inhibition as

$$\begin{array}{c} \gamma \propto 1-(\frac{I}{E}), \end{array}$$ (33)

which is confirmed in Fig 6D.

For Dalean matrices we used the SOC algorithm, with non-negative excitatory weights equal to $w_{0}/\sqrt{N}$ and inhibitory weights equal to $-(\frac{I}{E})w_{0}/\sqrt{N}$. Non-negative connections were randomly assigned with probability *p* from a uniform distribution, and thus the total excitatory (*E*) and inhibitory (*I*) input weights per neuron are

$$\begin{array}{c} E_{i}=\sum_{j=1}^{N/2}w_{ij}^{\text{B}}\approx E=\frac{Npw_{0}}{2\sqrt{N}}\text{,}\;\text{and}\;I_{i}=-\sum_{j=\frac{N}{2}+1}^{N}w_{ij}^{\text{B}}\approx I=-(\frac{I}{E})\frac{Npw_{0}}{2\sqrt{N}}. \end{array}$$ (34)

The initial weight, *w*_0_, is based on the eigenspectrum’s radius, *R*_outer_ (Eq 16), which results in the outlier being given by

$$\begin{array}{c} \gamma \approx \frac{pw_{0}\sqrt{N}}{2}[1-(\frac{I}{E})]=\frac{R_{\text{outer}}p[1-(\frac{I}{E})]}{\sqrt{\frac{2p(1-p)}{N}[1+(\frac{I}{E}{)}^{2}]}}. \end{array}$$ (35)

### Relationship between the eigenspectrum and inhibition-to-excitation ratio

In the networks with excitatory and inhibitory neurons, we enforce that inhibition dominates over excitation. This means that the variance of the weight distributions are different. In this case, the eigenvalues lie inside the circle of radius [2]

$$\begin{array}{c} R_{\text{outer}}\approx \sqrt{\frac{N}{2}(\sigma_{\text{E}}^{2}+\sigma_{\text{I}}^{2})}=w_{0}\sqrt{\frac{p(1-p)}{2}[1+(\frac{I}{E}{)}^{2}]}, \end{array}$$ (36)

where $\sigma_{\text{E}}^{2}$ and $\sigma_{\text{I}}^{2}$ are the variance of the excitatory and inhibitory weight distributions, respectively. Non-zero excitatory and inhibitory weights are equal to $w_{0}/\sqrt{N}$ and $-(\frac{I}{E})w_{0}/\sqrt{N}$, respectively, and randomly chosen with probability *p* drawn from a uniform distribution.

The distribution of eigenvalues is not uniform when the variance of excitatory and inhibitory weight distributions are different [2]. In this case there is an accumulation of eigenvalues inside an inner circle of radius given by $R_{\text{inner}}\approx \sqrt{N}\sigma_{\text{min}}$, where *σ*_min_ = min(*σ*_E_, *σ*_I_). When inhibition is stronger than excitation, the distribution with smaller variance is the excitatory one, and thus $\sigma_{\text{min}}^{2}=\sigma_{\text{E}}^{2}=w_{0}^{2}p\left(1-p\right)/N$, resulting in an inner radius given by

$$\begin{array}{c} R_{\text{inner}}\approx \sqrt{N}\sigma_{\text{min}}=w_{0}\sqrt{p(1-p)}=\frac{R_{\text{outer}}}{\sqrt{\frac{1}{2}[1+(\frac{I}{E}{)}^{2}]}}. \end{array}$$ (37)

## Supporting information

S1 Text Supplemental text. Sections “Why upper triangular?”, “Alternative feedforward structures”, and “Biologically plausible network dynamics with strictly positive rates”.
(PDF)

S1 Fig Imaginary clustering at different points. Dynamical responses of a spectrum that is clustered around the points 100 and −100 with respect to the imaginary axis. The imaginary radius around these points is 0.5. The real distribution is uniform on the interval (−0.5, 0.5). **A**, Linear dynamical response; the network shows an amplified response, effectively due to superposition of almost identical frequencies. Inset: eigenspectrum distribution. **B**, Nonlinear responses are not amplifying; the very high frequency together with the saturation point prevents the network’s modes from driving each other in order to create an amplifying response. **C**, Clustering at other finite points, {±10, ±30, ±50, ±70, ±90}, shows the same discrepancy between the linear and nonlinear behaviour, as measured by the percentage of amplified responses.
(EPS)

S2 Fig Varying the real and feedforward distributions. **A**, Exploring the single valued real distribution. We compare results for three real values: −0.5, 0 and 0.5. Top: maximum response norm for preferred initial condition. Naturally a larger real part leads to more amplification as the decay envelope becomes slower. Middle: % of amplified conditions that are amplified by at least 50%; this is also affected by the value of the real part, indicating that the amplification landscape changes its shape in a uniform way. Bottom: the percentage of pairwise eigenvector angles is independent of the real value, i.e., the increased amount of amplification is mainly a result of the slower decay times. Results in all cases are qualitatively similar in their dependence on the imaginary radius. **B**, Comparing results for two different feed-forward structures. One is the feedforward structure taken from the corresponding feedforward part of a matrix constructed using the Stability-Optimised algorithm [8] (pink). The other has a uniform feedforward entry distribution, with overall feedforward norm equal to the stability-optimised one (yellow). In both cases the spectra are identical and correspond to the spectral distribution of the pink curve from A, i.e., single real value at zero, varying imaginary range represented on the x-axis. **C**, Same as panel A but the real part of the eigenspectrum is uniformly distributed between (0.5 − *d*_re_, 0.5). Values of *d*_re_ are indicated in the figure (colour coded).
(EPS)

S3 FigEffective rank of matrix P for a larger number of principal components.**A**, Comparison of the number of columns and effective rank of the dynamical matrix P as a function of the imaginary diameter. The feedforward structure is random from a uniform distribution, and scaled to have Frobenius norm equal to 75. Number of neurons, *N* = 200. **B**, Number of columns and effective rank of P as a function of the feedforward norm. The imaginary diameter is fixed and equal to 20. Number of neurons, *N* = 200. The matrix P is constructed such that for each amplified initial condition **a**_**i**_ in $I_{B}$, there are *κ*_*i*_ principal components stored as columns in the matrix P. The number *κ*_*i*_ is the number of principal components that are needed to capture at least 85% of the total variance of the dynamical response given condition **a**_**i**_. The large discrepancy between the number of columns and the effective rank in the long transient regimes verifies the results shown in the manuscript.(EPS)

S4 FigComparison between upper triangular and recurrent matrices.**A**, Effective rank of the eigenvector matrix, **V**, for an upper triangular matrix (dashed cyan line) and a recurrent matrix (pink solid line) as a function of the imaginary diameter (left) and feedforward norm (right). The recurrent weight matrix, **W**_rec_, is the upper triangular matrix, $\tilde{\text{W}}$, rotated with an orthonormal basis **U**: ${\text{W}}_{\text{rec}}=U\tilde{\text{W}}U^{†}$. **B**, Same as panel A, but the effective rank is calculated for the weight matrix. **C**, Amplified directions and effective rank of the matrix P in the linear and nonlinear cases for the recurrent matrix in panels A and B as a function of the imaginary diameter (left) and the feedforward norm (right). The feedforward structure is random from a uniform distribution, and the real distribution is uniform on (−0.5, 0.5). In all cases the network size is *N* = 200. The feedforward Frobenius norm is fixed at 75 for the plot varying the imaginary diameter. The imaginary diameter is fixed at 20 for the plot with varying feedforward norm.(EPS)

S5 Fig Timescale of the transient dynamics as a function of the eigenspectrum’s imaginary diameter (left) and the feedforward norm (right) of an upper triangular matrix. Timescale is defined as the period, Δ*t*, for which ‖**r**(*t*)‖ ≥ 1 for the nonlinear network. The feedforward structure is random from a uniform distribution, and the real distribution is uniform on (−0.5, 0.5). The network size is *N* = 200, and the feedforward Frobenius norm is fixed at 75 for the plot varying the eigenspectrum’s imaginary diameter (left). The imaginary diameter is fixed at 20 for the plot with varying feedforward norm (right).
(EPS)

S6 Fig Regimes of transient amplification for distinct eigenspectra and feedforward structures from simulations (A-F) and analytics (G-I). **A-F**, Timescale of response (time for which ||**r**(*t*)‖ ≥ 1) for distinct eigenspectra and feedforward structures (specified below) from simulations with *N* = 200 neurons. **A**, Random uniform eigenspectrum distribution and random uniform feedforward distribution. Same plot as in Fig 5B. **B**, Eigenspectrum’s real part fixed at zero, random uniform distribution of the eigenspectrum’s imaginary part, and random uniform feedforward distribution. **C**, Inhibitory dominance in the eigenspectrum and random uniform feedforward distribution. **D**, Random uniform eigenspectrum distribution and feedforward distribution from stability-optimised circuit (SOC). **E**, Eigenspectrum’s real part fixed at zero, random uniform distribution of the eigenspectrum’s imaginary part, and feedforward distribution from SOC. **F**, Purely real eigenspectrum with inhibitory dominance and feedforward distribution from SOC. **G-I**, Normalised inner product of two eigenvectors from a 3-by-3 upper triangular matrix (Eq 4) as a function of feedforward norm and imaginary diameter for *α* − *γ* = 0 (**G**), *α* − *γ* = 128 (**H**), and *α* − *γ* = 1024 (**I**).
(EPS)

S7 Fig Effect of varying the outlier in the upper triangular setting without the zero trace condition. Maximum response norm for the preferred initial condition (left), percentage of directions whose norm is amplified more than 50% (middle), and percentage of angles, between pairs of eigenvectors, that are less than 45° (right) as a function of the imaginary diameter. In all cases the network size is *N* = 200 and the Frobenius norm of all matrices is normalised to 100. Different colours correspond to four different outlier values (colour coded). The rest of the eigenspectrum’s real distribution is the same: uniformly distributed between −0.5 and 0.5.
(EPS)

S8 Fig Feedforward (left) and spectrum (right) norms as a function of excitatory weights and *I*/*E* ratio in Dalean matrices without self loops. All matrices were generated with the SOC algorithm [8]. The y-axis corresponds to the value of individual excitatory weights, which are sparsely connected with probability 0.1 (see Methods). The x-axis corresponds to the *I*/*E* ratio, set to be constant by the SOC algorithm. In all cases the network size is *N* = 200 (100 excitatory and 100 inhibitory neurons), and the Frobenius norm of all matrices is normalised to 100.
(EPS)

S9 Fig Eigenvector overlap comparison. We compare the eigenvector overlap statistics as a function of the imaginary diameter. On one hand we compute the percentage of pairs that form angles < 45°. This is defined using the real part of the inner product, i.e., cos(*θ*) = Re(〈*α*, *β*〉), for the complex eigenvectors *α*, *β* (green). Alternatively, we can compute the percentage of pairs for which the magnitude of the complex–valued inner product |〈*α*, *β*〉| is larger than 0.7 (pink). Both yield similar results qualitatively.
(EPS)

S10 Fig Comparing different feedforward structures. Maximum response norm for the preferred initial condition (left), percentage of directions whose norm is amplified more than 50% (middle), and effective rank of the eigenvector matrix (right), as a function of the imaginary diameter. Three feedforward structures are compared. Green: uniform distribution as in Fig 3A of manuscript. Purple: a feedforward structure limited to chains of length 2, i.e., each Schur unit only connects directly to the next unit. Yellow: a sparse feedforward structure with probability of connection equal to 0.1. For normalisation reasons, the Frobenius norm of all feedforward structures is set to be equal to 75. Moreover, the real distributions of the sparse and 2-chain matrices do not satisfy the zero–trace condition. Inset in panel A shows the uniform and sparse networks again on a more appropriate scale for their values.
(EPS)
