# Supplementary material for: Regimes and mechanisms of transient amplification in abstract and biological neural networks
Source: PLoS Comput Biol. 2022 Aug 15;18(8):e1010365. doi: 10.1371/journal.pcbi.1010365 (PMC9377633; doi:10.1371/journal.pcbi.1010365)
Supplement: S1 Text — Sections “Why upper triangular?”, “Alternative feedforward structures”, and “Biologically plausible network dynamics with strictly positive rates”. (PDF) [file pcbi.1010365.s001.pdf]

Regimes and mechanisms of transient amplification in abstract and biological neural networks

Georgia Christodoulou[1], Tim P. Vogels[1,2,◕], Everton J. Agnes[1,3,◕,*],

1 Centre for Neural Circuits and Behaviour, University of Oxford, Oxford, United Kingdom
2 Institute of Science and Technology Austria, Klosterneuburg, Austria
3 Biozentrum, University of Basel, Basel, Switzerland

◕These authors contributed equally to this work.
* everton.agnes@gmail.com

Supporting information

S1 Text

Why upper triangular?

The idea behind the use of an upper triangular matrix arises from the real Schur decomposition. Given a connectivity matrix $\mathbf{W}$, one can find the eigenspectrum using the basis of eigenvectors. However, the non-normality of the matrix is lost under this linear transformation. Since we are especially interested in the dynamical regime of transient amplification we have to go beyond the spectrum, and a better way to access the non-normality is to use its Schur decomposition. Indeed, any square matrix is unitarily equivalent to an upper triangular one, and by definition, the minimum norm of the strictly upper part over all such decompositions is its non-normality index. We follow the same idea, but use instead the real Schur transformation. The advantage is that we still have in our hands a real-valued matrix. The disadvantage is that we now have to deal with 2×2 blocks along the diagonal. However, it is important to note that $\mathbf{W}$ is still orthogonally equivalent to its real Schur transform. This means that the non-normality quantity we are interested in is still preserved, i.e., the dynamical characteristics of transient amplification between the two matrices are not qualitatively different.

Alternative feedforward structures

In the main manuscript, the feedforward structure was taken to be dense and either random, or compatible with the feedforward structure of a stability-optimised circuit. To check how much our findings depend on those assumptions, we compare two significantly different feedforward structures (S10 Fig). The first is a sparse feedforward structure with probability of connection equal to 0.1. The second structure is comprised of feedforward chains limited to length 2. The simulations show that the results, as a function of the imaginary diameter, do not change qualitatively. However, in the case of the length 2 chains we see that the network is able to amplify much more. Intuitively, this effect might be explained by the fact that all structures are set to have equal norm. Consequently, the weights of the length 2 chains have very strong weights creating a long, strongly coupled and fully connected feedforward chain. Gradually increasing the length of the chains, produces gradually less amplification (not shown) since the weights

become weaker. The sparse network also has strong weights for the same reason. However, in this case the feedforward chain is sparse and disconnected, yielding less overall amplification.

Biologically plausible network dynamics with strictly positive rates

For mathematically tractability, we considered a linear input/output function to describe continuous firing-rate fluctuations of the network's neurons. For a more realistic comparison, we used a non-linear input/output function with a sigmoidal form (Eq. 2) with bounds defined by $r_{\min}$ and $r_{\max}$. In both cases, we considered the output, $\mathbf{r}(t)$, as the deviation from the baseline $\mathbf{r}_0$, and negative values would indicate rates below the baseline. A simple additive transformation can be used so that the network activity is strictly positive. Considering $\mathbf{r}(t) = f(\mathbf{x}(t)) + r_{\min}$, we can rewrite Eq. 1 as

$$\tau \frac{\mathrm{d}\mathbf{x}(t)}{\mathrm{d}t} = -\mathbf{x}(t) + \mathbf{W}\left[f\left(\mathbf{x}(t)\right) + r_{\min}\right] + \mathbf{h},$$

where $\mathbf{h}$ is a constant external input onto the network given by

$$h_i = -r_{\min} \sum_j W_{ij}.$$

The external input $\mathbf{h}$ can be interpreted as the baseline input that the neurons receive from upstream areas, which maintains the network's neurons firing at the baseline rate, $\mathbf{r_0}$.
